# A central cavity within the holo-translocon suggests a mechanism for membrane protein insertion

**DOI:** 10.1038/srep38399

**Published:** 2016-12-07

**Authors:** Mathieu Botte, Nathan R. Zaccai, Jelger Lycklama à. Nijeholt, Remy Martin, Kèvin Knoops, Gabor Papai, Juan Zou, Aurélien Deniaud, Manikandan Karuppasamy, Qiyang Jiang, Abhishek Singha Roy, Klaus Schulten, Patrick Schultz, Juri Rappsilber, Giuseppe Zaccai, Imre Berger, Ian Collinson, Christiane Schaffitzel

**Affiliations:** 1 European Molecular Biology Laboratory, Grenoble Outstation, 71 Avenue des Martyrs, 38042 Grenoble, France; 2 School of Biochemistry, University of Bristol, BS8 1TD, United Kingdom; 3 Department of Integrated Structural Biology, Institut de Génétique et de Biologie Moléculaire et Cellulaire (IGBMC), U964 INSERM, UMR7104 CNRS; University of Strasbourg, 1 Rue Laurent Fries, BP10142, 67404 Illkirch, France; 4 Wellcome Trust Centre for Cell Biology, University of Edinburgh, Edinburgh, EH9 3JR, United Kingdom; 5 Department of Physics, University of Illinois Urbana Champaign, 3217 Beckman Institute, 405 N Mathews Ave., Urbana, IL 61801, USA; 6 Department of Bioanalytics, Institute of Biotechnology, Technische Universität Berlin, 13355 Berlin, Germany; 7 Institut Laue Langevin, 71 Avenue des Martyrs, F-38042 Grenoble, France; 8 CNRS, Institut de Biologie Structurale, F-38044 Grenoble, France

## Abstract

The conserved SecYEG protein-conducting channel and the accessory proteins SecDF-YajC and YidC constitute the bacterial holo-translocon (HTL), capable of protein-secretion and membrane-protein insertion. By employing an integrative approach combining small-angle neutron scattering (SANS), low-resolution electron microscopy and biophysical analyses we determined the arrangement of the proteins and lipids within the super-complex. The results guided the placement of X-ray structures of individual HTL components and allowed the proposal of a model of the functional translocon. Their arrangement around a central lipid-containing pool conveys an unexpected, but compelling mechanism for membrane-protein insertion. The periplasmic domains of YidC and SecD are poised at the protein-channel exit-site of SecY, presumably to aid the emergence of translocating polypeptides. The SecY lateral gate for membrane-insertion is adjacent to the membrane ‘insertase’ YidC. Absolute-scale SANS employing a novel contrast-match-point analysis revealed a dynamic complex adopting open and compact configurations around an adaptable central lipid-filled chamber, wherein polytopic membrane-proteins could fold, sheltered from aggregation and proteolysis.

The hetero-trimeric Sec protein-conducting channel translocates integral inner membrane proteins and secretory proteins into or across the membrane^1^,^2^. Doung and Wickner discovered that in bacteria additional factors associate with this complex to facilitate efficient protein translocation and named the supercomplex “preprotein translocase holoenzyme”^3^. They co-immunoprecipitated SecYEG, YajC and SecDF as well as a ~60 kDa protein which was subsequently identified as YidC^4^ from radiolabeled *Escherichia coli* membranes using an anti-SecG antibody. Further study, however, was impeded by the lack of means to produce holo-translocon in the quality and quantity required for its biochemical and structural characterization. More recently, using recombinant highly purified SecYEG-SecDFYajC-YidC holo-translocon, it was shown that the complex is active in co- and post-translational translocation^5^. A recent proteomics study in *E. coli* based on absolute protein synthesis rates provided protein copy number estimates^6^ (Figure S1). This data is consistent with a molar ratio of SecY, SecE, SecG, SecD, SecF, YajC and YidC of ~4:4:4:1:1:10:3 in the membrane, suggesting that as much as ~25% of all SecYEG could be complexed in HTL.Under optimal growth conditions, the protein synthesis of HTL could amount to 2,600 copies of HTL per generation^6^; the real copy number is likely smaller, since this number does not take into account any protein turnover. Even accounting for a high level of turnover, this is in stark contrast to a previous copy number estimation based on semi-quantitative alkaline phosphatase-SecDF fusion protein analyses^7^ suggesting that *E. coli* membranes contain only ~10–30 copies of SecDF and about ~10-times more SecYEG copies.

The SecYEG core-translocon forms a central pore through which hydrophilic polypeptides are transported, otherwise closed by a girdle of hydrophobic residues and a short helix (plug)^8^. A lateral gate is formed between transmembrane helices (TMs) 2b and 7 of SecY through which TMs partition into the lipid bilayer. YidC is required to facilitate this passage from the lateral gate and for the subsequent folding and assembly of inner membrane-proteins and complexes^4^,^9^,^10^,^11^,^12^,^13^. Crystal structures of YidC show a large periplasmic domain and a conserved bundle of 5 TMs containing a hydrophilic groove at the cytosolic face for substrate binding and for facilitating membrane traversal^14^,^15^. The ancillary sub-complex comprising SecD and SecF stimulates protein translocation through SecYEG^8^ assisted by the transmembrane proton-motive force (PMF)^16^,^17^. The periplasmic domain of SecD consists of a P1-head and a P1-base domain, which are thought to contact the substrate and move in response to proton translocation; thereby facilitating the passage of polypeptides across the membrane^17^.

Here, we present an interdisciplinary analysis of HTL architecture combining small-angle neutron scattering (SANS), electron microscopy (EM) and biochemical and biophysical data in an integrated approach. Absolute-scale contrast variation SANS revealed a dynamic HTL complex and a lipid-filled central cavity surrounded by protein. The surrounding protein components were then visualized by cryo-EM. Their identities and arrangement were further characterized by EM analyses of HTL sub-complexes, with missing components. The data and available crystal structures of the individual subunits enabled us to build a quasi-atomic model of the complex, which lends itself to an interesting new mechanism for membrane protein insertion.

## Results

### HTL comprises one copy each of its subunits

For balanced over-production of the functional bacterial HTL complex we used the ACEMBL expression system^18^, which allowed HTL isolation by detergent solubilisation, affinity purification via the hexahistidine-tags fused to SecE, SecD and YidC and the calmodulin-binding peptide fused to YajC, followed by gradient centrifugation (Supplementary Fig. S1)^5^. Size-exclusion chromatography and analytical ultracentrifugation of the detergent-solubilized HTL are compatible with a protein complex of a molecular weight of ~250 kDa comprising one copy each of the subunits *plus* contributions from lipids and detergent (Supplementary Fig. S1c,d)^5^. The detergent-solubilized complex was expected to be highly dynamic with respect to its periplasmic domains, which are flexibly linked to the transmembrane parts. Moreover, the HTL complex is sensitive to increased detergent concentrations^5^. Importantly, HTL cannot be reconstituted from its individual components. Therefore, we applied mild glutaraldehyde cross-linking to produce stable particles suitable for further analysis, or we exchanged the detergent DDM (n-Dodecyl-β-D-Maltopyranoside) with amphipols. It is important to note that crosslinking cannot ‘induce’ a new conformation, it will stabilize existing conformations of a protein complex. Glutaraldehyde reacts preferentially with amine groups and has a variable linker length. As other crosslinking reagents, glutaraldehyde will therefore stabilize preferentially rather compact conformations of a dynamic complex compared to more open conformations.

### Small-angle neutron scattering reveals a dynamic HTL complex with a lipid-filled cavity

In order to explore the component composition and conformation of HTL, we performed SANS on the native HTL complex as well as a mildly cross-linked version of it. The cross-linking procedure was developed to reduce the expected flexibility and to increase the stability of the complex, in aid of subsequent analysis by EM (see below). Through analysis of the D_2_O/H_2_O contrast match points, we can determine the composition of our HTL sample with respect to protein and non-protein components (lipids and detergent). Moreover, we can determine the location of these components relative to one another. Thus, SANS allows an independent, model-free dissection of the HTL three-component system.

**Figure 1:**
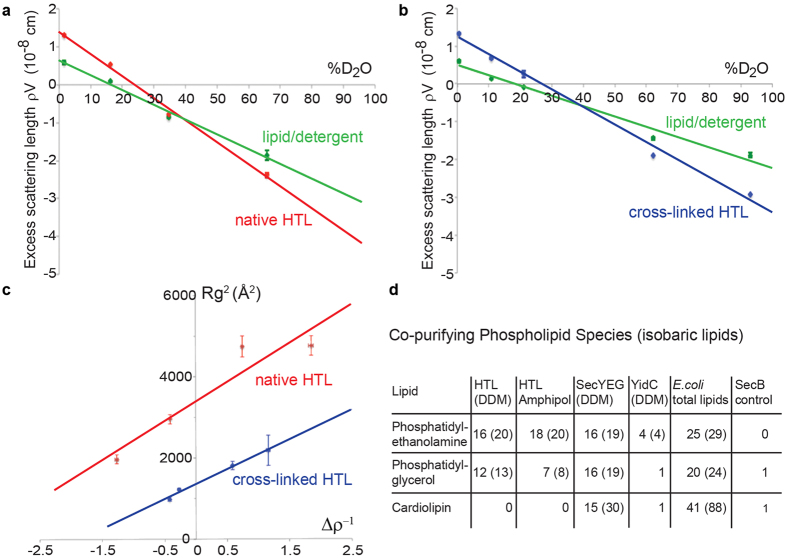
SANS Analysis reveals a dynamic HTL with a lipid-filled cavity. (**a**,**b**) Excess scattering length ρV of native HTL (**a**) and cross-linked HTL (**b**) at different D_2_O concentrations and resultant calculated scattering lengths of HTL’s lipid/detergent component. Error bars represent the error in the I(0) determined from the Guinier analysis. (**c**) Stuhrmann plot for native and cross-linked HTL. The square of the radius of gyration (R_g_) is plotted against the inverse of the particles’ neutron scattering contrast Δρ. Error bars in the y-direction represent the errors in R_g_ determined from the Guinier analysis, and in x-direction the effect of labile hydrogen-exchange varying between 70% and 90%. The 36% data point for cross-linked HTL is not shown because of the large error associated with the R_g_ due to the protein component being nearly matched out. (**d**) Table summarizing the distinguishable lipid species identified by mass spectrometry in HTL preparations (DDM-solubilized and in amphipols), SecYEG, YidC and in control samples (*E. coli* total lipids and the cytoplasmic protein SecB). The numbers in bracket include the isobaric lipid species, i.e. these numbers take into account that several lipids in *E. coli* have an identical mass/charge ratio and therefore cannot be distinguished by MS. The numbers in the table thus indicate how many different lipids were detected, and the numbers in brackets indicate the maximal number of species that could be present in the sample. We note that cardiolipin is present in the control preparations of SecYEG while it is not present or lost in HTL purifications. The false discovery rate in this analysis is very low, since in independent analyses of cytoplasmic SecB (negative control) one or no lipid species were matched.

Absolute-scale SANS collected at different D_2_O concentrations were interpreted by a novel contrast match point (CMP) analysis. The procedure enabled the dissection of the contribution of the protein, lipid and detergent components to the HTL structure based on their respective chemical composition and partial specific volume (CMP = 39.0%, 21.7%, 13.1% D_2_O, respectively, for protein, DDM and lipid). The lipid CMP was calculated from the lipid composition of the inner *E. coli* membrane. The measured CMP of the native HTL complex (24 ± 0.2% D_2_O) indicated a substantial contribution from non-protein components. The protein component contribution was calculated and subtracted from the complex contrast variation line to yield the CMP of the non-protein component (16.5 ± 0.3% D_2_O), allowing the lipid/DDM volume ratio to be calculated. The SANS data indicates that the composition of native and cross-linked HTL in DDM is consistent and mono-disperse, with single copies of each protein subunit. Importantly, the match-point analysis (Fig. 1a, Supplementary Fig. S2, Supplementary Table S1) indicates the native HTL complex isolated in DDM consists of not only the 250 kDa protein component (33 ± 1% v/v) in detergent (26 ± 2% v/v), but also contains a large proportion of lipids (41 ± 3% v/v), equating to 330–390 lipid molecules per HTL complex. Corresponding values for the mildly cross-linked HTL complex are similar for protein and detergent (41 ± 2% and 33 ± 2% v/v), but containing fewer lipids (25 ± 3% v/v, equivalent to 160–200 lipid molecules) (Fig. 1b, Supplementary Fig. S2, Supplementary Table S1). Taken together, these data suggest that lipids form an integral part of the HTL complex, remaining protected from the detergent during extraction from the bacterial membrane.

Furthermore, SANS analysis allowed determining the location and dimensions of the protein and lipid/detergent constituents of HTL^19^. Stuhrmann^20^ has shown that by changing contrast in SANS, using different solvent H_2_O/D_2_O mixtures, the scattering curve can be interpreted mathematically in terms of the scattering density distribution within the particle. In the so-called Stuhrmann plot, the square of the radius of gyration is plotted versus inverse contrast (see Methods). A straight line plot indicates that the centres of mass of the density distribution and equivalent homogeneous particle coincide. A positive slope indicates higher density further away from the centre, while a negative slope indicates higher density close to the centre. A parabolic plot indicates a separation between the centres of mass of particle shape and density distribution. In cross-linked HTL the radius of gyration of lipid/detergent (R_g_-lipid/detergent) is 22 ± 4 Å, while that of protein (R_g_-protein) is 52 ± 5 Å. The calculated R_g_ protein of the EM-reconstruction is ~44 Å (see below) and thus in reasonable agreement with our SANS R_g_-protein, considering the different sample states. The positive slope of the Stuhrmann plot (Fig. 1c) clearly indicates that the higher-scattering density proteins surround an inner core of lower-scattering density lipids. The native HTL-complex had an equivalent structural organization, but appears to be significantly larger (R_g_-lipid/detergent = 49 ± 2 Å; R_g_-protein = 75 ± 3 Å). In conclusion, the SANS data imply that the HTL forms a highly dynamic complex with an extended, open conformation predominant in DDM and a more compact arrangement stabilized by cross-linking. Lipids are an integral part of the HTL complex and are located in the centre surrounded by proteins, as indicated by the smaller radius of gyration of lipids compared to protein.

### Nature of the lipids in the HTL preparations

The presence of lipids in HTL, implicated by SANS, was further confirmed by mass spectrometry. We asked whether a particular lipid species would co-purify with the HTL preparations. The predominant phospholipids of the *E. coli* inner membrane, phosphatidyl-ethanolamine and phosphatidyl-glycerol, were identified in HTL complexes isolated in DDM and amphipols (Fig. 1d). Thus, the lipid species co-purifying with HTL are representative of those found in the surrounding *E. coli* inner membrane.

### Electron microscopy locates YidC and SecYEG within the HTL

**Figure 2:**
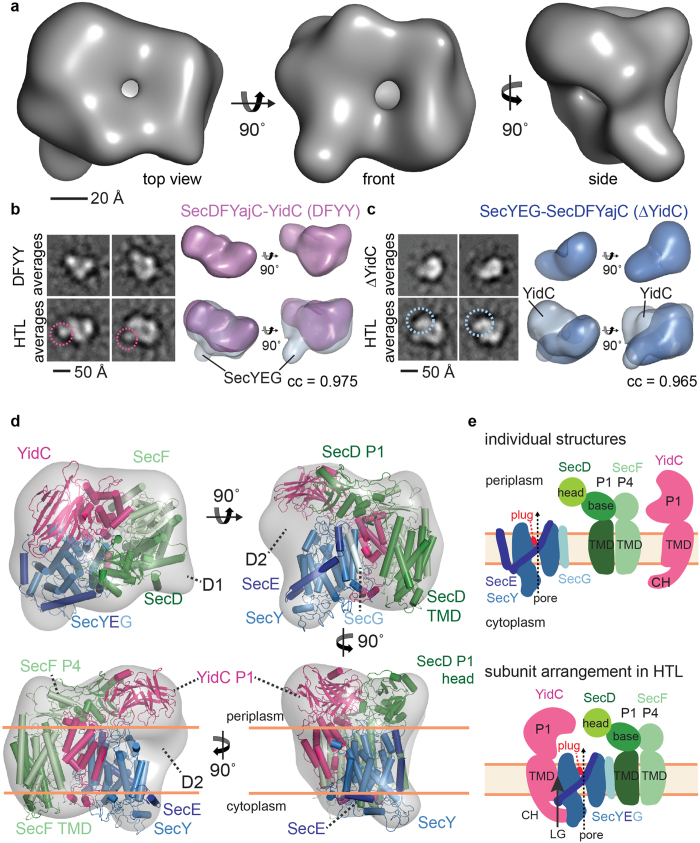
Electron microscopy of HTL and subcomplexes. (**a**) *E. coli* holo-translocon cryo-EM reconstruction displayed in a top (left), front (middle) and side view (right). (**b**) Localization of SecYEG in HTL. Left: comparison of reference-free 2D class-averages of DFYY and HTL (pink circle: density attributed to SecYEG). Right: RCT reconstruction of DFYY (purple), below: Superimposition of DFYY and HTL (transparent gray) reconstructions (correlation coefficient (cc) 0.975). (**c**) YidC localization. Left: comparison of reference-free 2D class-averages of ΔYidC and HTL (blue circle: density attributed to YidC). Right: RCT reconstruction of ΔYidC (blue), below: Superimposition of ΔYidC and HTL reconstructions (cc of 0.965). (**d**) Fitting of crystal structures (YidC) and homology models of SecYEG and SecDF into HTL density (transparent grey), shown in a top (top left), front (top right) and two side views (below). Periplasmic domains of YidC, SecD and SecF are labelled P1 and P4 respectively. Unaccounted density is labelled D1 and D2. (**e**) Scheme of HTL assembly. SecY is colored marine, SecE dark-blue, SecG cyan, SecD green, SecF light-green, and YidC magenta.

Due to the flexibility of the complex (see above), mild glutaraldehyde cross-linking was deployed in order to produce stable, homogeneous particles suitable for analysis by EM. An initial HTL volume was determined using negative-stain EM and random conical tilt (RCT) reconstruction (Supplementary Fig. S3). Subsequently, cryo-EM 2D class-averages (Supplementary Fig. S4) were aligned against the RCT reconstruction to generate a cryo-EM volume. 3D maximum likelihood refinement using 53,648 particles yielded a low-resolution (14 Å) structure (‘gold-standard’ method^21^ and Fourier shell correlation (FSC) criterion 0.143^22^, Supplementary Fig. S4c,d) with dimensions of 11.4 nm × 10.6 nm × 9.2 nm (Fig. 2a). The handedness of the reconstruction was confirmed by tilt pair validation (Supplementary Fig. S4e,f).

In parallel, we performed cryo-EM of native HTL complex stabilized by amphipols (Supplementary Fig. S5). The resulting reconstruction of this uncross-linked HTL converged at a similar resolution (15 Å) (‘gold-standard’ refinement^21^ and FSC criterion 0.143^22^, Supplementary Fig. S5d) and shows correspondent features to the mildly cross-linked version. The compaction observed in the un-crosslinked HTL-amphipol sample may originate from loss of detergents and lipids during purification and exchange of detergent with amphipols (see Methods). A striking feature of the visualised HTL is that the protein complex is hollow. Indeed, the SANS data indicates that the interior contains lipids, which we cannot detect by EM at this resolution.

The consistency we observed between the shapes of uncross-linked HTL-amphipol and cross-linked HTL-DDM is noteworthy at this intermediate resolution and compelled us to perform subunit localization by analysing stable HTL sub-assemblies missing individual subunits. We calculated RCT reconstructions for SecYEG-SecDF-YajC (ΔYidC) and SecDF-YajC-YidC (DFYY)^23^ sub-complexes (Supplementary Fig. S1, Supplementary Fig. S3b,c) and superimposed them with the HTL RCT volume (Fig. 2b,c). The locations of SecYEG and of the YidC P1-domain were verified in 2D class-averages of the three complexes (Fig. 2b,c). Therefore, we placed an *E. coli* homology model of SecYEG and the *E. coli* YidC structure^15^ as rigid bodies into the respective EM density of the HTL (Fig. 2d,e, Supplementary Fig. S6). The EM volume representing SecYEG is somewhat reduced when compared to the crystal structure, indicative of flexibility in this region of HTL. Our intermediate resolution EM volumes of the HTL super-complex provided useful boundary conditions to model HTL architecture: The placement of SecYEG and YidC defined the orientation of the HTL complex, localizing the transmembrane (SecYEG) and periplasmic domains (YidC-P1).

### Flexibility of the SecD periplasmic domain

**Figure 3:**
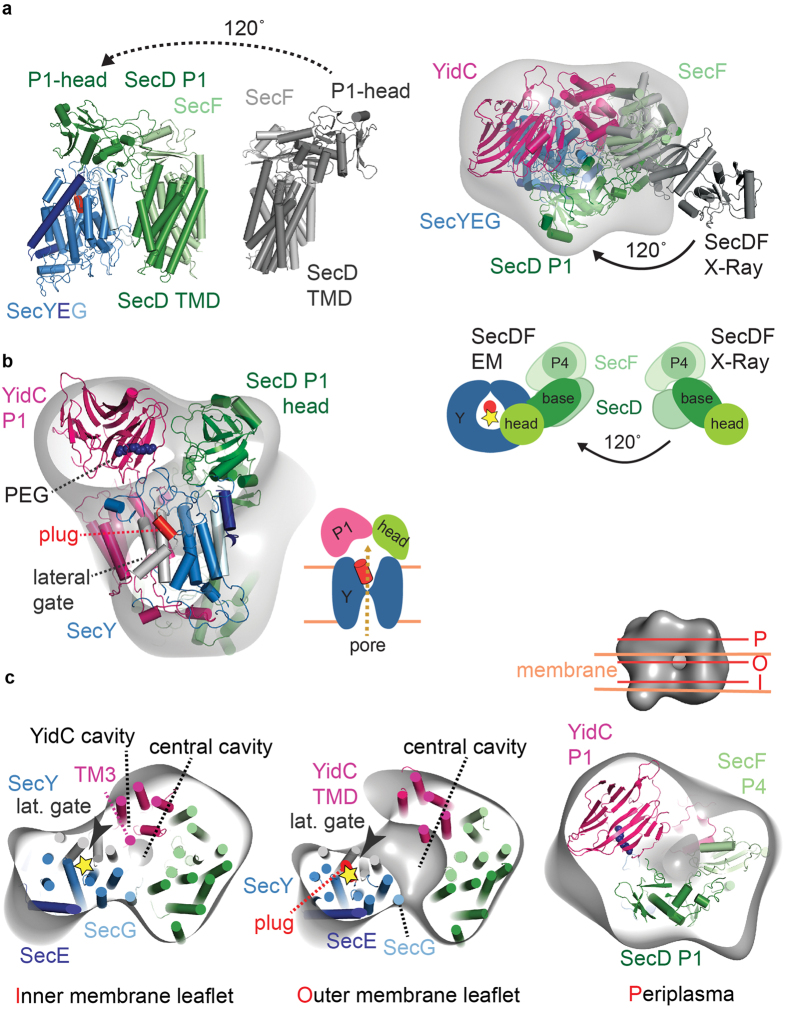
Domain arrangement in the HTL complex. (**a**) Conformational change of the SecD periplasmic domain placing the P1-head domain above the translocation channel. Comparison (above) and overlay (below) of the SecDF homology model based on the cryo-EM density (green and light-green) and the *Th. thermophilus* SecDF crystal structure^17^ (grey). (**b**) The HTL positions the periplasmic domains of SecD and YidC for substrate binding. The YidC P1 and SecD P1-head domains are located above the SecY translocation-pore sealed by the plug (red) in the inactive state. YidC P1 has a polyethylene-glycol (PEG) molecule (violet spheres) bound in a putative hydrophobic substrate-binding cleft^28^. (**c**) HTL horizontal sections (red lines) in the periplasmic region (right), outer membrane leaflet (middle) and inner membrane leaflet (left), highlighting the ring-like arrangement of the periplasmic domains, the positioning of YidC next to the lateral gate formed by SecY TM-helices and the position of the water-accessible, intramembrane cavity of YidC in our model, and the existence of a central cavity in HTL. Color coding as in Fig. 2; SecY lateral gate-helices are grey in panel (**b**,**c**); the position of the SecY translocation-pore is marked with a yellow star.

A homology model of the SecDF sub-complex was placed as a rigid body into the remaining density (Fig. 2d), with the exception of the SecD P1-domain. Best fit of P1 was achieved independently from the transmembrane part and the SecF periplasmic domain (P4), by rotation of ~120° (Fig. 3a). In the crystal structure from *Th. thermophilus*, the SecD P1-base and the SecF P4-domain adopt ferredoxin-like folds^17^, forming apparently continuous antiparallel β-sheets across the SecD-SecF interface. However, careful inspection reveals that the P1–P4 interface interaction is weak with only a single hydrogen-bond present (Supplementary Fig. S7a). Thus, it is conceivable that the periplasmic domains of SecD and SecF can flexibly disengage from one another to adopt the conformation found in HTL. This flexibility of the SecD P1-domain is further supported by recent EM reconstructions of *Th. thermophilus* SecDF^24^. Moreover, the region linking TM-helix1 to the P1-domain of SecD is not conserved between *Th. thermophilus* and *E. coli* (Supplementary Fig. S7b). Notably, the rotated SecD periplasmic domain is compatible with all the EM reconstructions presented here. The rotated version of the SecDF subcomplex fits into the native HTL-amphipol cryo-EM reconstruction as well as in the negative stain EM reconstructions of cross-linked HTL, SecDFYajC-YidC and of ΔYidC complexes (Supplementary Fig. S7c).

YajC is part of the HTL complex, but its function remained enigmatic. When we include YajC in the HTL model at the proposed position^25^ it would be bound at the interface of SecDF facing the YidC TM domain and SecG (Supplementary Fig. S8).

### SecD and YidC periplasmic domains are positioned above the translocation pore exit site of SecY

The quasi-atomic model corresponding to 75% of HTL (Supplementary Fig. S6b) was flexibly-fitted into the map employing the molecular dynamics flexible-fitting method and NAMD2.10, coupling the Cα-chain to the map^26^. EM density, not filled by the atomic model, is observed next to the SecD P1-domain (‘D1’ in Fig. 2d), likely due to the fact that the *E. coli* P1-domain is larger than the corresponding *Th. thermophilus* P1-domain on which our model is based (Supplementary Fig. S7b). Likewise, unassigned density is observed next to SecYEG in the transmembrane region (‘D2’ in Fig. 2d) potentially corresponding to two additional N-terminal TMs of SecE and/or TM1 of YidC of which atomic coordinates are not available.

Importantly, in the HTL the three periplasmic domains of SecD, SecF and YidC adopt a ring-like arrangement, wherein the SecD P1-head and YidC P1 are positioned at the exit site of the SecY translocation channel (Fig. 3b,c). This interpretation is consistent with previous studies demonstrating cross-links between SecD and SecY/SecE/SecG^5^, and interactions between the P1-domain of YidC and SecG/SecD/SecF^12^,^27^. This arrangement could enable these large periplasmic domains to contact emerging polypeptides and facilitate their translocation (Fig. 3b), potentially also aided by the PMF^28^,^29^.

### The YidC transmembrane domain contacts SecY and SecF

The HTL model is fully compatible with previous functional analyses. For instance, in the HTL membrane-sector, SecG is positioned close to the TM-domain (TMD) of SecD (Fig. 3c), consistent with known genetic interactions^3^,^30^. Close-by the SecY lateral gate, density attributed to the conserved TM domain of YidC extends to YidC P1-domain (Fig. 3c). The resulting proximity of the YidC TMD to SecDF is supported by recent independent screening and complementation experiments^31^. The YidC TMD was suggested to facilitate membrane-protein insertion, borne out by cross-linking between nascent TM-helices successively to SecY, lipids and finally to YidC^10^, including YidC-TM3^32^.

Additional cross-links between the cytoplasmic C-terminus of YidC to SecF and SecY (but not to SecD) support the placement of the YidC TMD within the HTL^12^ (Supplementary Table S2). Similar to SecYEG, the density corresponding to YidC is somewhat reduced indicating flexibility (Fig. 3c), a feature also apparent in the crystal structure^14^. In the inner membrane leaflet, towards the cytoplasmic face of the HTL, there is an inter-connecting density between YidC and SecY, highlighting an interaction^12^ (Fig. 3c). Evidently then, YidC is well-positioned within the HTL to chaperone the entry of TM-helices emerging from the lateral gate of SecYEG into the lipid bilayer, possibly using the water-accessible YidC cavity as a first binding site for emerging TM-helices^14^,^15^,^33^. The horseshoe-shape arrangement of the TM-domains of the HTL encloses the central cavity within the protein complex (Fig. 3c), which is occupied by phospholipids as evidenced by our SANS analyses.

## Discussion

Membrane protein biogenesis and protein secretion are fundamental cellular processes. In particular membrane-protein folding and complex assembly is poorly understood because it is challenging to study experimentally. We previously described the generation of an expression system for simultaneous production of all seven subunits of the *E. coli* holo-translocon complex^18^. The concomitant over-expression of all seven constituents of the HTL complex allowed the extraction and isolation of the intact complex in a mild detergent, DDM^18^. Subsequent biochemical characterization showed that the HTL preparations are active in co-translational membrane protein translocation and in post-translational protein secretion^5^. HTL was shown to be less active in protein secretion compared to SecYEG, but pro-OmpA secretion was stimulated much more effectively by the PMF in HTL, likely due to the presence of SecDF^5^. Importantly, HTL is more active in insertion and assembly of many membrane protein substrates, compared to SecYEG or YidC alone^5^,^34^. Recent proteomics data from *E. coli* indicate that about 25% of SecYEG could be part of HTL complexes^6^. In agreement, HTL can be immuno-precipitated from native *E. coli* inner membranes using anti-SecG antibodies after membrane treatment with mild detergents^3^ or with SMALPs (styrene maleic acid lipid particles)^34^. Taken together, we suggest that HTL has an important role in supporting membrane protein integration and folding as well as complex formation^5^,^34^. It is reasonable to assume that HTL is not required for all translocation processes and that a large number of translocation substrates can be translocated by SecYEG alone, for instance secreted proteins which appear to be less efficiently translocated through HTL^5^. In light of these assumptions, which still have to be challenged experimentally, we consider ~25% of SecYEG in HTL complexes a very reasonable and significant number.

**Figure 4:**
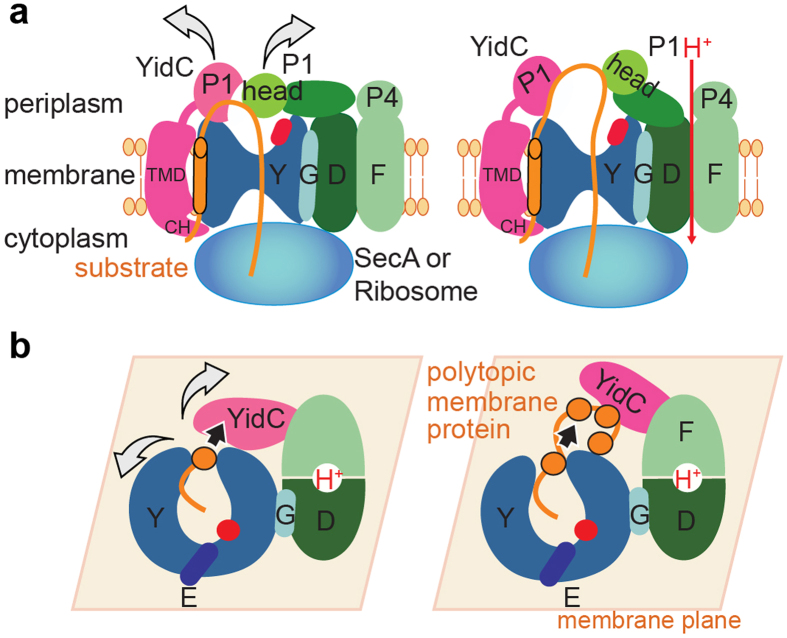
Mechanistic model of protein translocation catalyzed by the holo-translocon. (**a**) Protein translocation through SecYEG driven by the PMF and SecDF. The YidC periplasmic and SecD P1-head domains are positioned to interact with translocation substrates preventing backsliding of the polypeptide through the translocation channel. Translocation is additionally energized by SecA ATPase or the translation machinery. (**b**) Membrane-protein integration and folding can occur at the interface between the SecY lateral gate and YidC in a protected lipid-HTL environment where TM-helices are suggested to accumulate until they can fold into a structured domain.

To explain the mechanism of HTL-conducted protein translocation^5^, it is essential to understand the functional interplay of SecYEG, SecDF-YajC and YidC during translocation. While crystal structures of SecYEG, SecDF and YidC are available^8^,^14^,^15^,^17^,^35^, the subunit organization in the HTL complex remained unclear. Here, we combined a range of biophysical data to provide mechanistic and structural insights into the HTL membrane protein complex in an integrative approach. Based on the SANS data, EM volumes, subunit localization experiments and available biochemical data^3^,^4^,^5^,^12^,^27^,^31^ we present a quasi-atomic model of HTL and a working model for HTL function in translocation into and across the membrane and membrane protein folding (Fig. 4).

Importantly, the architecture of HTL reveals that SecD and YidC P1-domains are positioned above the translocation pore formed by SecY. Both domains have previously been suggested to interact with translocation substrates^28^,^29^. Thus, in HTL SecD and YidC P1-domains are optimally positioned to bind and prevent backsliding of translocation substrates (Fig. 4a). YidC P1 contains an elongated cleft, which in the crystal structure was occupied with a PEG molecule and therefore suggested to bind unfolded non-polar translocated peptides^28^. The interaction of the SecD P1-head domain with translocation substrates is suggested to induce conformational changes in the SecDF complex, coupling protein translocation with proton transport across the membrane^17^, thus using the PMF to facilitate translocation. In agreement, we previously showed that proOmpA secretion by HTL is stimulated by the PMF^5^. Consistent with this, the immobilization of SecD upon antibody contact inhibits protein secretion, causing the accumulation of precursors *in vivo*^29^.

Surprisingly, we identified a lipid-filled cavity within the HTL-complex by SANS and EM. We propose that it provides an adaptable, protected environment for membrane-protein insertion, folding and controlled release into the inner membrane (Fig. 4b). Such a mechanism is analogous to those deployed by molecular chaperones such as Trigger-Factor and GroEL, which provide hydrophilic encapsulated environments for folding and then release of globular proteins. With this in mind we suggest the HTL has a chaperone function beyond the activity of YidC alone. In our model, the membrane-protein chaperone YidC assists in folding by binding successive TMs exiting from the SecY lateral gate (Fig. 4b). In HTL, an opening for the release of TM-bundles could then be achieved by YidC flexibly moving away from SecYEG, reminiscent of an airlock with two sealable gates. Similar mechanisms may also operate in the eukaryotic translocation-complex where membrane-proteins are suggested to partition in the lipid bilayer and fold in the vicinity of the Sec61 protein-conducting channel, the translocation-associated membrane-protein (TRAM) and additional associated complexes^36^; this study supports a conserved mode of action. The results presented highlight the intricate and highly dynamic structure of HTL and give crucial new insights into the functional interplay between SecYEG, SecDF, YidC and phospholipids, providing a framework for our understanding the universal process of membrane-protein insertion.

## Methods

### Expression and purification of holo-translocon complex and subcomplexes

The expression plasmid pACEMBL_HTL3 was generated using the ACEMBL expression system^5^,^18^. The ACEMBL acceptor and donor plasmids were combined by Cre-LoxP fusion to yield pACEMBL_DFYY. pACEMBL_HTL3ΔYidC was generated by deletion of the YidC encoding gene from the pACEMBL_HTL3 expression plasmid. HTL and SecDF-YajC-YidC (DFYY) were expressed in BL21star(DE3) (Invitrogen) and affinity purified as described via His_6_-tags fused to the N–terminus of SecE, the C–terminus of SecD, the C–terminus of YidC and via the CBP-tag fused to the C–terminus of YajC^5^ with the following modifications: The Ni-NTA affinity-purified protein was transferred into CBP buffer (50 mM Hepes-KOH, 130 mM NaCl, 10% glycerol, 2 mM CaCl_2_, 0.03% DDM, pH 8.0) using a desalting column (GE Healthcare) and loaded onto a calmodulin affinity column (Stratagene). HTL was eluted with CBP elution buffer containing 2 mM EGTA. For electron microscopy and biophysical characterization, the complexes were stabilized by mild glutaraldehyde cross-linking^37^. 1.5 mg (2 ml) HTL was loaded onto a 40 ml glycerol gradient from 10% to 30% glycerol and from 0 to 0.15% glutaraldehyde in 20 mM Hepes-KOH, 130 mM NaCl, 5 mM Mg(OAc)_2_, 0.03% DDM, pH 8.0 and centrifuged for 36.5 h at 83,000 × g and 4 °C (SW32 rotor, Beckman Coulter). The fraction (1 ml) containing HTL was supplemented with 100 μg of lysine and concentrated using a concentrator with a molecular weight cut-off (MWCO) of 30 kDa (Amicon). The concentrated fraction was further purified using a Superose6 column (GE Healthcare) equilibrated with HSM buffer (20 mM Hepes-KOH, 130 mM NaCl, 2 mM Mg(OAc)_2_, 0.03% DDM, pH 8.0). pACEMBL_HTL3ΔYidC was expressed in BL21star(DE3) (Invitrogen) and purified as described above. In the purified HTL3ΔYidC complex, a band corresponding to YidC in the complex could not be detected, neither in the Coomassie-stained SDS gel nor by Western blot using a mouse anti-YidC antibody, which recognizes the YidC periplasmic domain (Supplementary Fig. S1) in agreement with a very strong overexpression of SecYEG-SecDFYajC compared to the endogenous levels of YidC.

For HTL-amphipol reconstitution, the CBP-affinity purified HTL was transferred in desalting buffer (20 mM Hepes-KOH, 130 mM NaCl, 5% glycerol, 0.03% DDM, pH 8.0) using a disposable desalting column (Bio-Rad). A8-35 amphipols (Anatrace) were added to the protein solution at a protein:amphipol mass ratio of 1 to 4 and incubated under gentle agitation for 2 hours at 4 °C. In the meantime, biobeads (Bio-Rad) were washed for 30 minutes in methanol under gentle agitation followed by three washes in milliQ water for 5 minutes. The detergent molecules were removed from the solution by adding biobeads to the protein solution at a detergent:biobead mass ratio of 1 to 20 and incubated overnight at 4 °C under gentle agitation. The protein/amphipols solution was recovered by pipetting and subjected to ultracentrifugation for 30 minutes at 47,000 × g and 4 °C (TLA55 rotor, Beckman Coulter). Subsequently, the sample was concentrated using a concentrator with a molecular weight cut-off (MWCO) of 30 kDa (Amicon) and subjected to size exclusion chromatography using a Superdex200 column (GE Healthcare) equilibrated with 20 mM Hepes-KOH, 130 mM NaCl, 5% glycerol, pH 8.0.

### Analytical ultracentrifugation

Absorbance at 280 nm and interference profiles were measured for 16 h at 35,000 rpm and 10 °C in a Beckman XL-I analytical ultracentrifuge with an An-60Ti rotor with 12 mm optical path length cell equipped with sapphire windows. Data were analysed as described^38^. A frictional ratio of 1.3 and a partial specific volume of 0.76 (intermediate between that of the protein complex with an expected molecular mass of 250 kDa and that of the detergent) were used. Sample density and viscosity were 1.007 g/ml and 1.35 mPa·s, respectively, as determined with Sednterp.

### Random conical tilt reconstructions

Holo-translocon, DFYY and ΔYidC (~0.15 mg/ml each) were absorbed to a carbon-coated electron microscopy grid for 30 s, and negatively stained with 1% uranyl acetate for 30 s. A total of 300 micrographs were recorded at room temperature on a BioTwin CM120 electron microscope (FEI) at a magnification of 71,550× and a defocus of ∼2 μm at 120 kV, using a 4 k × 4 k CCD camera. Two consecutive images of the same area were taken at 45° and 0° tilt angles under low-dose conditions. The contrast transfer function (CTF) was analyzed and corrected using BCTF (Bsoft package ref. 39). 11 HTL, 9,908 DFYY and 10,986 ΔYidC tilt pairs were selected manually using Tiltpicker^40^. Untilted images were aligned using iteratively refined 2D class averages as references and were subjected to multivariance statistical analysis (MSA) and Hierarchical Ascendant Classification for clustering into 400 class-averages with IMAGIC-5^41^. 400 volumes were calculated from the 2D classes and were aligned, averaged and clustered using Xmipp MLtomo^42^ to compensate for the missing cone, resulting in 10 RCT reconstructions for each sample. The two most populated volumes of each sample were subjected to further refinement cycles through projection matching with Spider^43^, additionally including 15,000 untilted images for HTL, 13,465 for DFYY and 11,473 for ΔYidC. For all samples, the two volumes converged during refinement. For subunit mapping, the 3D volumes of HTL, DFYY and ΔYidC were superimposed using Chimera (http://www.cgl.ucsf.edu/chimera).

### Electron Cryo-Microscopy

Purified HTL (0.15 mg/ml) was applied to a thin carbon foil sustained by a holey carbon grid (Quantifoil 2/2) and plunge-frozen in liquid ethane with controlled temperature and humidity (Vitrobot, FEI). Samples were imaged under low-dose conditions (~10 e^−^ Å^−2^) at a magnification of 78,000× on a Polara cryo-transmission electron microscope (FEI) at 100 kV. 2,500 micrographs were recorded on a 4 k × 4 k direct electron detector (Falcon I, FEI) at 1.36 Å/pix and coarsened three times resulting in a final pixel size of 4.08 Å. HTL-amphipols were plunge-frozen using holey-carbon grids (Quantifoil 2/2) and imaged on a Titan Krios (FEI) equipped with a 4 k × 4 k direct electron detector (Falcon II) at a pixel size of 1.01 Å using 36 electrons per Å^2^. We also collected data at 300 kV, but we then could not identify the particles in the vitreous ice with confidence.

### Image Processing

The CTF was determined using Bsoft^39^, and the image phases were flipped accordingly. Using EMAN2^44^, 84,732 particles were boxed, subjected to 2D MSA and classification and compared to projections of the refined HTL RCT volume. At this step, 300 2D class-averages corresponding to 23,749 particles of crosslinked HTL and 35,521 particles of HTL-amphipols were rejected based on visual inspection and lower correlation with the projections from the RCT model. The RCT volume was used to assign the projection angles to the remaining 800 2D class-averages of HTL and 1,000 class-averages of HTL-amphipols to calculate the 3D reconstruction from the cryo-EM data with the BP RP program of Spider^43^. The resulting initial cryo-EM volumes contained 60,983 HTL particles and 119,346 HTL-amphipol particles. For the HTL-amphipol sample, 3D classification resulted in one class with improved features (Supplementary Fig. S5f), which was subjected to maximum likelihood 3D refinement until convergence in Relion^45^. The final reconstructions of crosslinked HTL and of HTL-amphipols after gold-standard refinement^21^ contained 53,648 and 23,381 particles respectively. After high-resolution noise substitution^46^, the resolution of the structures was 14 Å for the cross-linked HTL and 15 Å for HTL-amphipols, based on the Fourier shell correlation criterion 0.143^22^ (Supplementary Figs S4c, S5d).

### Tilt Pair Validation

To confirm the handedness of the map, HTL and 70 S ribosome samples were negative stained and imaged on the same electron microscope at 0° and 10° tilt angles, using the same magnification and detector. Particles were manually picked in e2RCTboxer, and aligned to the random-conical-tilt reconstruction of HTL (Supplementary Fig. S3) and the cryo-EM map of a 70 S ribosome^47^ respectively using Relion. Dots were plotted in e2tiltvalidate^44^ with filtering to exclude those with out-of-plane rotation larger than 30°. The position of each dot represents the direction and the amount of tilting for a particle pair in polar coordinates. For both samples dots cluster in the same direction of the plot at a tilt angle of approximately 10°. This confirms that our HTL map has the correct handedness (the same as the 70 S ribosome map).

### Generation of the quasi-atomic model of the HTL

Homology models of SecD and SecF were generated based on the crystal structure of *Th. thermophilus* SecDF^17^ using ClustalW2 (http://www.clustal.org/clustal2/, and the Swiss-Model workspace (http://swissmodel.expasy.org/). The SecYEG homology model is based on the *Methanococcus jannaschii* SecYEβ structure^8^ which represents an inactive translocon where the channel is sealed with a plug. Moreover, we used the *E. coli* YidC crystal structure for model building^15^. The structures were placed into the EM map with Chimera (http://www.cgl.ucsf.edu/chimera) and Pymol (DeLano Scientific). For SecD, the periplasmic domain was fitted independently from the TMDs (Fig. 3a). Interactive fitting of atomic structures was performed using molecular dynamics flexible-fitting simulations at 300 K^48^,^49^. Only Cα atoms were coupled to the density, in vacuum, with a g-scale of 0.3. The simulation was run for 10 ns till RMSD from the initial structure converged to 5 Å and the global correlation coefficient increased to 86%. The resulting atomic model is embedded into a 3POPE: 1POPG membrane, solvated, ionized.

### Lipid/detergent density in the HTL

Our SANS experiments (Fig. 1a) unambiguously demonstrate that a large fraction of lipids and DDM (~60% v/v) is present in the lipid-filled cavity and surrounding the protein. Notwithstanding, when we analysed the very same sample by cryo-EM, we did not detect the lipids in the cryo-EM density at the current resolution, neither as positive nor as negative density. We attribute this to flexibility. In fact, the EM density volumes for SecYEG and the TM domain of YidC appear smaller than the corresponding protein volume, indicating flexibility. We hypothesize that the lipid and detergent in the central cavity and surrounding the HTL may be likewise flexible and lack the order required for visualization at the current resolution.

### Lipid analysis by mass spectrometry

#### Samples preparation

800 pmol of five samples named as YidC, SecYEG, SecB, detergent-solubilized HTL (DDM) and HTL in amphipol A8-35 (HTL-amphipol) were dissolved in 1 ml of HEPES buffers (pH 8.0). 0.03% DDM was added to YidC, SecYEG and HTL (DDM). 3.5 ml of chloroform:methanol solution (1:2 v/v) was added to the samples and agitated for 30 seconds. Subsequently, 0.5% acetic acid in 500 mM NaCl was added (to increase the yield of phospholipids) and agitated for 30 seconds. Finally 1.25 ml of chloroform was added and agitated for 30 seconds. Finally, the samples were centrifuged in swing-out centrifuge for 10 minutes at 1200 rpm. 2 ml of the bottom layer comprising the lipids were collected and transferred into a new glass tube. The extraction was evaporated in a desiccator under vacuum for 3 hours at room temperature.

#### Mass spectrometry

Samples were resuspended in the 200 μl 50/50 (v/v) acetonitrile/water for 10 min at room temperature and subsequently centrifuged for 10 mins at 13,000 rpm. The supernatant was analyzed on a LTQ-Orbitrap Velos mass spectrometer (Thermo Fisher Scientific) coupled with Dionex Ultimate 3000 RSLC nano system. The column with a spray emitter (75-μm inner diameter, 8-μm opening, 160 mm length; New Objectives) that was packed with C18 material (ReproSil-Pur C18-AQ 3 μm; Dr Maisch GmbH, Ammerbuch-Entringen, Germany) using an air pressure pump (Proxeon Biosystems). The mobile phase A consisted of 50/50 (v/v) acetonitrile/water. Mobile phase B consisted of 9/10 (v/v) isopropanol/acetonitrile. 0.05% (v/v) ammonia solution was added into both buffer A and B. Samples were loaded onto the column with 100% A at 500 nL/min flow rate and eluted at 300 nL/min flow rate with linear gradient increase from 65% B to 98% B in 29 minutes; then keep 98% B for 15 minutes. The eluted phospholipids were directly sprayed into the mass spectrometer. The LTQ-Orbitrap Velos was operated in negative ion mode with spray voltage of 1.8 kV and capillary temperature of 240 °C. Full scan MS were acquired in the Orbitrap (m/z 350–2000) with a resolution of 100,000 and an automatic gain control (AGC) target at 1E6. The three most intense ions were selected for CID fragmentation in the ion trap and HCD in the Orbitrap with dynamic exclusion for 120 s. Ions with unrecognized charge state were excluded. CID normalized energy 38%, activation of q 0.25, activation time 30 ms, AGC 10,000; HCD normalized energy 65%, activation time 40 ms, AGC 50,000, resolution 7500.

#### Data Analysis

Data were analysed by LipidSearch software (version1.0, *Thermo* Fisher Scientific). Database: Orbitrap, search type: Product; Exp Type: LC-MS; Precursor tol: 5 ppm; Product tol: 0.2 Da; Intensity threshold, relative 1%; m-Score Threshold: 10.0. Class: PE, PG, CL; Ion: -H and -2H. Each sample was run 4 times. All the reported lipid ions were identified at least twice.

### Small angle neutron scattering of holo-translocon

#### Sample preparation

Native HTL was purified as described (ref. 5).

#### SANS data collection

HTL complexes were concentrated and buffer exchanged into the appropriate D_2_O buffers using a 50 kDa MWCO centrifugation filter (Millipore). An experimentally determined molar extinction coefficient of ε_HTL_ = 497,000 M^−1^ cm^−1^ was used to estimate protein concentration (c = 0.5 A mg.ml^−1^, where A is the absorbance at 280 nm for 1 cm pathlength). The protein complexes were stored at 4 °C and used within 48 h after gel filtration. Scattering data for the native HTL were collected on beamline D22 at the Institut Laue Langevin (ILL, Grenoble, France), using 6 Å wavelength, at two instrumental detector/collimator configurations, 2 m/2 m and 5.6 m/5.6 m. SANS data for the cross-linked HTL were collected on beamline KWS-2 at Jülicher Zentrum für Forschung mit Neutronen (JCNS, Garching, Germany), using 6 Å wavelength, at distances 1.67 m and 7.67 m with collimation at 4 m and 8 m, respectively.

The samples were measured in Hellma quartz cells 100QS with either 0.100 or 0.200 cm optical path. The temperature was kept between 4 and 8 °C. Scattering patterns were radially averaged and corrected for buffer scattering and detector responses using standard software from the ILL (ftp://ftp.ill.eu/pub/cs/sans/SansManual_and_2012update.pdf) and from JCNS (www.qtikws.de). The scattering data from H_2_O buffer was used to place the data on an absolute scale.

#### SANS data analysis

Guinier analysis of the data was accomplished using Igor Pro software (WaveMetrics, Lake Oswego, OR) with SANS macros developed at the NCNR (NIST, Gaithersburg, USA). The Guinier approximation straight line fit was used on the low-Q portions of the data to obtain values and errors for the radius of gyration R_g_ and the forward scattering intensity I(0) of the samples^19^. In general, the Guinier approximation is considered valid for q.R_g_ ~1. For certain globular shapes, however, the linear range can extend significantly further.

The linear dependence of the excess scattering length of the particle obtained from the square root of I(0) divided by concentration versus solvent scattering length density expressed as equivalent percentage of D_2_O (Fig. 1a,b) indicates that the scattering particle was constant for all D_2_O conditions measured—i.e. in both composition (protein-lipid-DDM stoichiometry) and association state (mild aggregation or polydispersity).

The scattering length 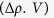 was determined by using the formula:


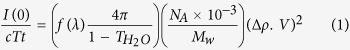


*c*: protein concentration in mg/ml; T: transmission of the sample, t: optical path length. The first bracket on the right represents normalisation by water. *f*(*λ*) for 6 Å wavelength is 0.8. Transmission of water (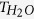) is 0.53. The second bracket converts mass concentration into number of particles. M_W_: molar mass of protein component; *N*_A_: Avogadro’s number.


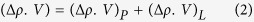



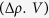
 is the sum fo protein and non-protein (lipid/detergent) component scattering lengths, respectively:

Equation (1) results in the scattering length of the complex at X% D_2_O is therefore proportional to √(I(0)/C at X% D_2_O. The theoretical scattering length of the protein component was calculated using MULCh^50^. The oligomeric state of the protein was determined directly from the 0% D_2_O data as 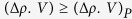. The molar mass of the protein component alone, calculated from the extrapolation to 0% D_2_O of √(I(0)/C) at the lipid/DDM match point (16.5% and 18% D_2_O respectively for the native and the cross-linked HTL) putting a zero value at 39% the match point of the protein, indicated that the HTL complex included one copy of each subunit.

Contrast match point Match_total_ for the particle occurs at D_2_O concentration 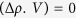. It is significantly lower than that of protein alone indicating the presence of a component (DDM/detergent mixture) of lower CMP. Incorporating into the above equations the experimental √(I(0)/C vs %D_2_O line permitted the determination of the volume and CMP of the lipid/DDM component. Correspondingly, the contrast match points for the protein and non-protein components of the particle were estimated as follows. Recasting formula (2) gives


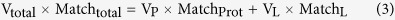


V_total_: volume of particle; V_P_: volume of protein; Match_P_: match point of protein. V_L_: volume of non-protein component; Match_L_: match point of non-protein component. V_total_ is the sum of V_P_ and V_L_. V_P_ was directly calculated from the protein sequence and thus the volumes of particle and non-protein component were derived from the scattering data.

A similar approach was pursued to determine the composition of the non-protein component of the particle. Contrast match points of detergent and lipids have been measured here (for DDM) and by others^19^. The lipid CMP was calculated from the lipid composition of the inner *E. coli* membrane. Note the contrast match points of proteins, detergent and lipids are significantly different (39.0%, 21.7% and 13.1%). We included an extra 5% error for the lipid/detergent scattering length due to estimating the level of labile Hydrogen-exchange for the protein component of HTL. Based on this, we estimated the proportion (v/v) of protein, lipid and detergent: 33 ± 1% protein, 41 ± 3% lipid, 26 ± 2% detergent for the native HTL, and 41 ± 2% protein, 25 ± 3% lipid, 33 ± 2% detergent for the mildly cross-linked sample used for EM.

Guinier-derived R_g_ values were used for the calculation of the Stuhrmann plot^19^,^20^. R_g_s determined from different D_2_O concentrations are related to contrast by 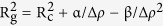 (Δ*ρ* is mean contrast for HTL; R_c_ is R_g_ at infinite contrast, α and β are scattering density related coefficients; α relates to the distribution of scattering densities relative to the center of mass; β provides the separation of the mass centers of the two components). The radius of gyration of the individual components of the HTL complex was determined by plotting the observed overall R_g_ against the inverse of the total contrast (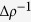). The observation that the data can be fit linearly (β ≈ 0) implies the centers of mass of the two components coincide within the accuracy of the measurements. Moreover, the positive slope of the fit at infinite contrast (Δ*ρ*^−1^ = 0) in Fig. 1b demonstrates that the higher contrast component (here protein) lies towards the periphery of the complex. Consequently, the HTL has a lipid/detergent core surrounded by protein.

The observed R_g_ can also be recast as follows:


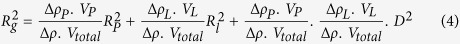


where 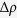 and V are for the protein (P), the non-protein components (L) and the total particle (total). D is the distance between the centers of mass of the protein and of the non-protein components. In the parallel axis analysis, if 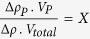 and therefore 
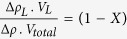
, formula (4) should be rewritten as:


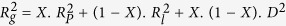


or more clearly,


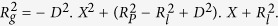


The observed R_g_ plotted against *X* would yield the same information as the Stuhrmann plot. Here however, at X = 1, the scattering will only be due to the protein component, and at X = 0, the scattering will only due to the non-protein component.

## Additional Information

**How to cite this article**: Botte, M. *et al*. A central cavity within the holo-translocon suggests a mechanism for membrane protein insertion. *Sci. Rep.* **6**, 38399; doi: 10.1038/srep38399 (2016).

**Publisher’s note:** Springer Nature remains neutral with regard to jurisdictional claims in published maps and institutional affiliations.

## Supplementary Material

Supplementary Information
