# Supplementary material for: A central cavity within the holo-translocon suggests a mechanism for membrane protein insertion
Source: Sci Rep. 2016 Dec 7;6:38399. doi: 10.1038/srep38399 (PMC5141469; doi:10.1038/srep38399)
Supplement: Supplementary Information [file srep38399-s1.pdf]

SUPPLEMENTARY INFORMATION

A central cavity within the holo-translocon suggests a mechanism for membrane protein insertion

Authors: Mathieu Botte[1,+], Nathan Zaccai[2,+], Jelger Lycklama à Nijeholt[1], Remy Martin[2], Kèvin Knoops[1], Gabor Papai[3], Juan Zou[4], Aurélien Deniaud[1], Manikandan Karuppasamy[1], Qiyang Jiang[1], Abhishek Singha Roy[5], Klaus Schulten[5], Patrick Schultz[3], Juri Rappsilber[4,6], Giuseppe Zaccai[7,8], Imre Berger[1,2], Ian Collinson[2,*], Christiane Schaffitzel[1,2,*]

Affiliations:

[1] European Molecular Biology Laboratory, Grenoble Outstation, 71 Avenue des Martyrs, 38042 Grenoble, France.

[2] School of Biochemistry, University of Bristol, BS8 1TD, United Kingdom.

[3] Department of Integrated Structural Biology, Institut de Génétique et de Biologie Moléculaire et Cellulaire (IGBMC), U964 INSERM, UMR7104 CNRS and University of Strasbourg 1 Rue Laurent Fries, BP10142, 67404 Illkirch, France.

[4] Wellcome Trust Centre for Cell Biology, University of Edinburgh, Edinburgh EH9 3JR, United Kingdom.

[5] Department of Physics, University of Illinois Urbana Champaign, 3217 Beckman Institute of Advanced Science and Technology, 405 N Mathews Ave., Urbana, IL 61801.

[6] Department of Bioanalytics, Institute of Biotechnology, Technische Universität Berlin, 13355 Berlin, Germany.

[7] Institut Laue Langevin, 71 Avenue des Martyrs, F-38042 Grenoble, France.

[8] CNRS, Institut de Biologie Structurale, F-38044 Grenoble, France.

[+] These authors contributed equally to this work.

[*] correspondence: christiane.berger-schaffitzel@bristol.ac.uk; ian.collinson@bristol.ac.uk.

Supplementary Figures

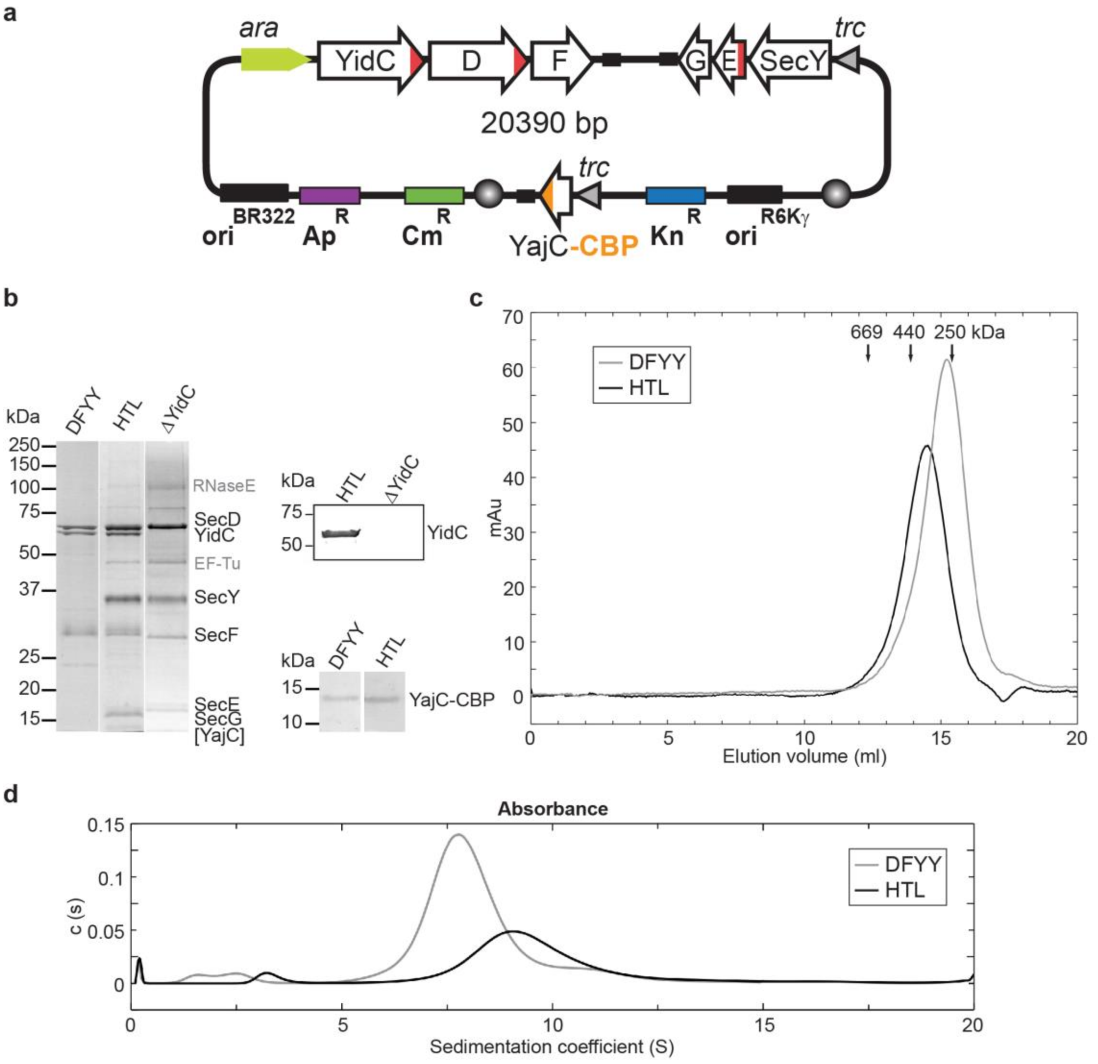

e

Gene	mRNA level (RPKM)	Translation efficiency (AU)	Synthesis rate (mRNA level·× transl. efficiency)	Stoichiometry (normalized)
secD	483	0.43	207.69	1.0
secF	466	0.48	223.68	1.1
secY	11006	0.07	770.42	3.7
secE	1252	0.64	801.28	3.9
secG	3452	0.27	932.04	4.5
yidC	882	0.77	679.14	3.3
yajC	1037	2	2074	10.0

Figure S1. Purification and biophysical characterization of holo-translocon and SecDF-YajC-YidC complexes. a, schematic representation of the pACEMBL_HTL3 expression plasmid containing a polycistron encoding for YidC, SecD (D), SecF (F) with His_6-tags fused C-terminally to SecD and YidC under control of an

arabinose promoter (ara), a second polycistron encoding for SecY, SecE (E) and SecG (G) with N-terminal His6-tag (red) fused to SecE under control of a trc promoter (trc) and calmodulin-binding protein (CBP, orange)-tagged YajC under the control of the trc promoter. Transcriptional terminators are shown as small black rectangles. Origins of replication (BR322 and R6Kγ) are indicated by large black rectangles. Antibiotic resistance genes confer resistance to the following antibiotics: Ap (ampicillin, purple), Cm (chloramphenicol, green), and Kn (kanamycin, blue). **b,** Coomassie-stained SDS-PAGE of holo-translocon (HTL), SecDF-YajC-YidC (DFYY) and SecYEG-SecDF-YajC (ΔYidC) complexes (left). Bands at ~100kDa and ~45kDa were identified by mass spectrometry as RNaseE and Elongation Factor Tu respectively. Western blot using a mouse anti-YidC antibody and an anti-mouse IgG antibody-alkaline phosphatase conjugate to confirm the absence of YidC in the ΔYidC sample (right, above), and detection of the CBP-tagged YajC protein by Western blotting using biotinylated calmodulin and a streptavidin-horseradish peroxidase conjugate (right, below). **c,** Superose6 size exclusion chromatography profile of HTL and DFYY. HTL elutes at an apparent molecular weight of ~360kDa, consistent with a molecular weight of ~250kDa for HTL protein components plus detergent and lipids. DFYY elutes at ~270kDa. The elution volume of proteins used for calibration is indicated by arrows. **d,** Analysis of sedimentation velocity of mildly cross-linked HTL and DFYY. The panel presents the absorbance distribution c(S). AUC data analysis indicates a particle of 350-400 kDa including ~0.5g DDM bound per 1g protein for 80% of the HTL sample and a 250-300kDa particle including ~0.5g DDM per 1g protein for 85% of the DFYY sample. In panels c and d, HTL is shown as a black line, DFYY as a grey line. **e,** *E.coli* mRNA levels and translation efficiency. The average mRNA levels (RPKM) and translation efficiency (arbitrary unit) for *E. coli* grown in MOPS complete medium are listed in black. In blue: calculated stoichiometries, normalized for SecD. [Data (black) taken from Table S4, Li et al., Cell 2014 (Suppl. Ref. 1)]

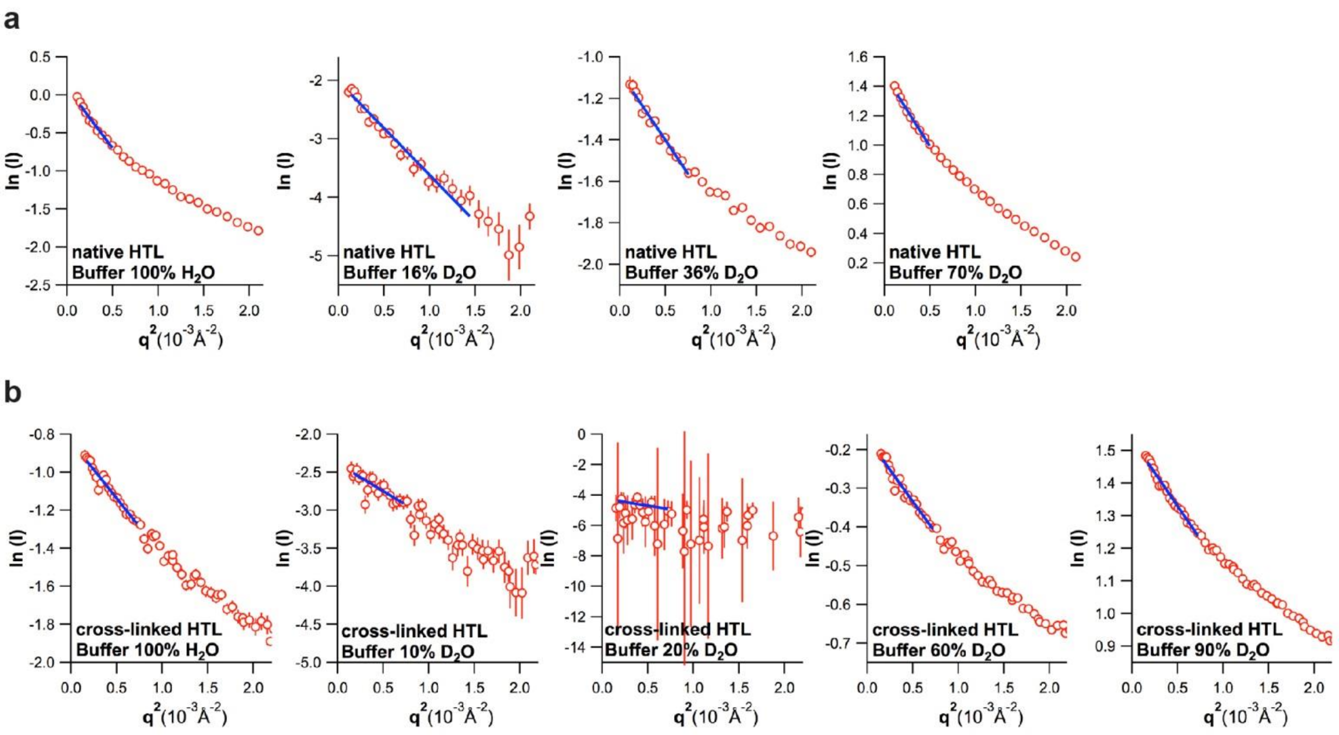

Figure S2. Guinier analysis. a, SANS data for native HTL. Guinier plots and linear fits in different D_2O buffers. **b,** SANS data for cross-linked HTL. Guinier plots and linear fits in different D_2O buffers.

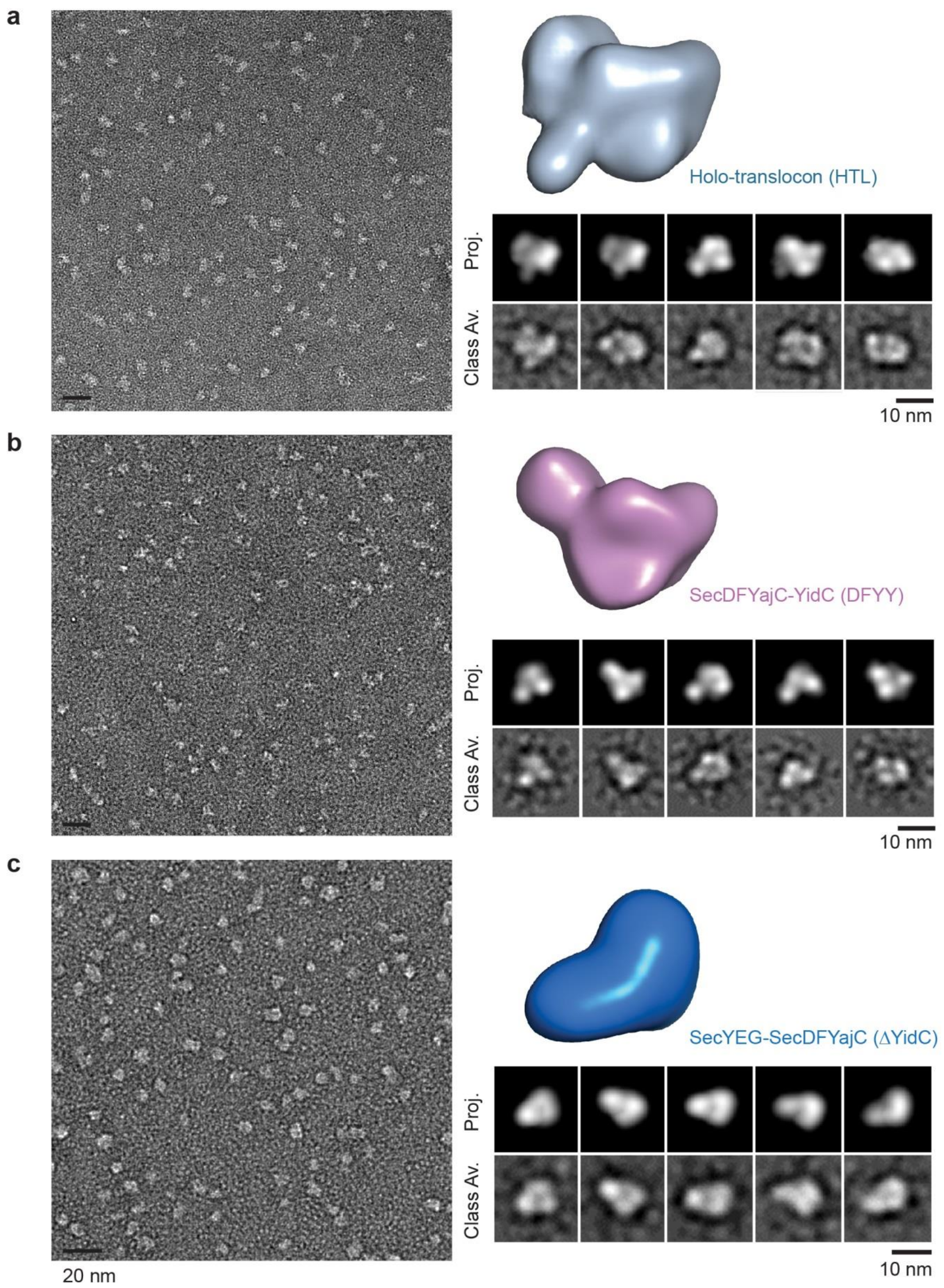

Figure S3. Random conical tilt (RCT) reconstructions of **a,** holo-translocon, **b,** SecDFYajC-YidC (DFYY) and **c,** SecYEG-SecDFYajC (ΔYidC). Each panel shows the negative-stain micrograph of the respective complex (left), the calculated RCT volume (top right), projections of the RCT volume (middle, right) and corresponding reference-free 2D class-averages of the negative-stain EM data (bottom, right).

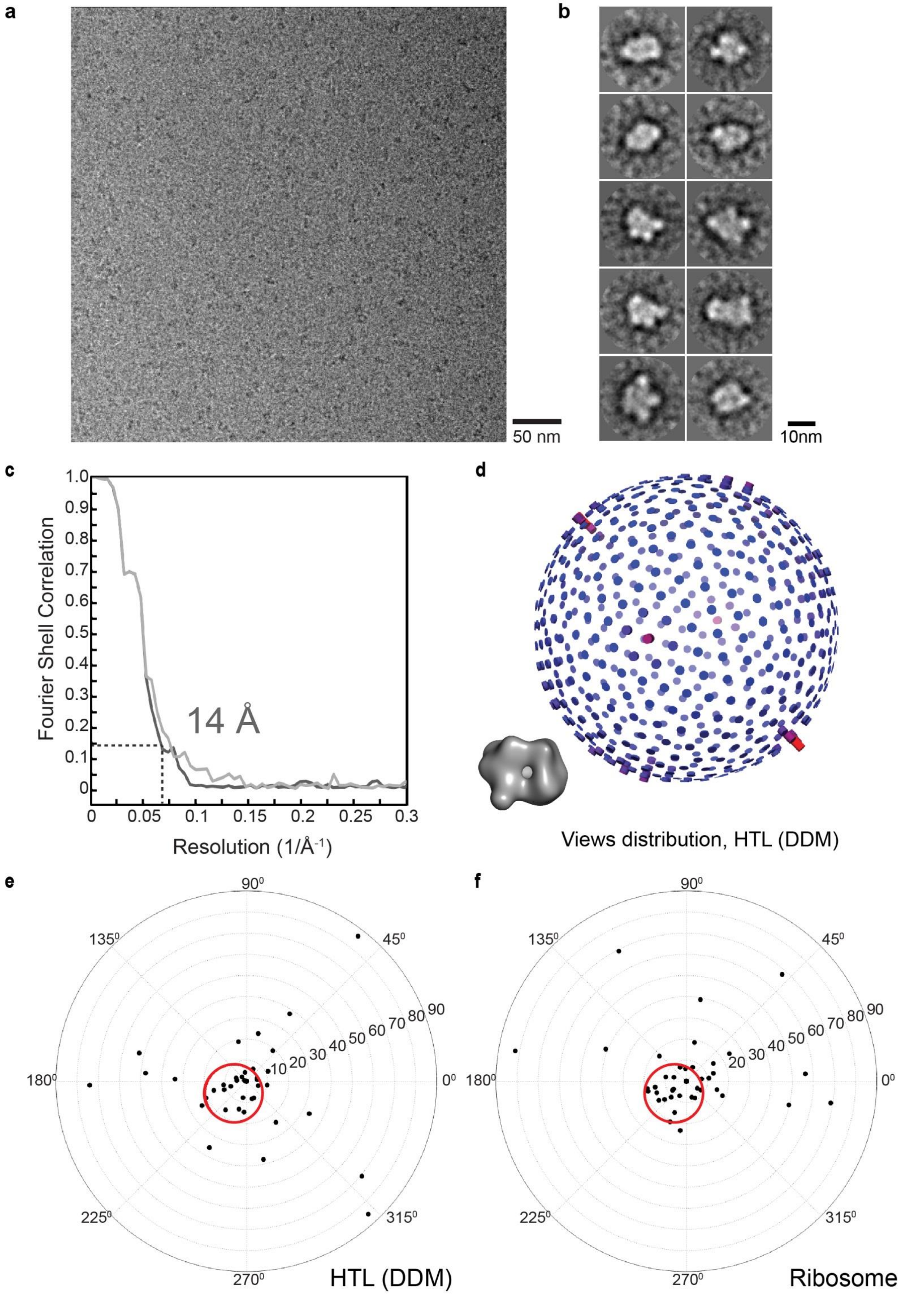
a
b
50 nm
10nm
c
Fourier Shell Correlation
14 Å
Resolution (1/Å⁻¹)
d
Views distribution, HTL (DDM)
e
HTL (DDM)
f
Ribosome

Figure S4. Cryo-EM of detergent-solubilized, mildly cross-linked HTL. a, Original micrograph of HTL embedded in vitreous ice taken at a magnification of 78,000×. **b,** Reference-free 2D class-averages of the cryo-EM data. **c,** Fourier Shell Correlation (FSC) curve of the cryo-EM HTL structure determined using the 'gold standard method' [2]. Curves are shown before (light grey) and after (dark grey) high-resolution noise substitution [3]. FSC=0.143 [4] indicates a resolution of 14 Å. **d,** Euler angle distribution of particles used for calculating the HTL cryo-EM structure (53,648 particles; angular step 8 degrees). The height of the cylinders represents the number of particles. **e,f,** Tilt-pairs of negative-stained, DDM-solubilized HTL (e) and of the *E. coli* 70S ribosome as a control for the handedness (f). For both samples the dots cluster in the same direction of the plot at a tilt angle of ~10°, confirming that our HTL map has the correct handedness i.e. the same as the 70S ribosome map.

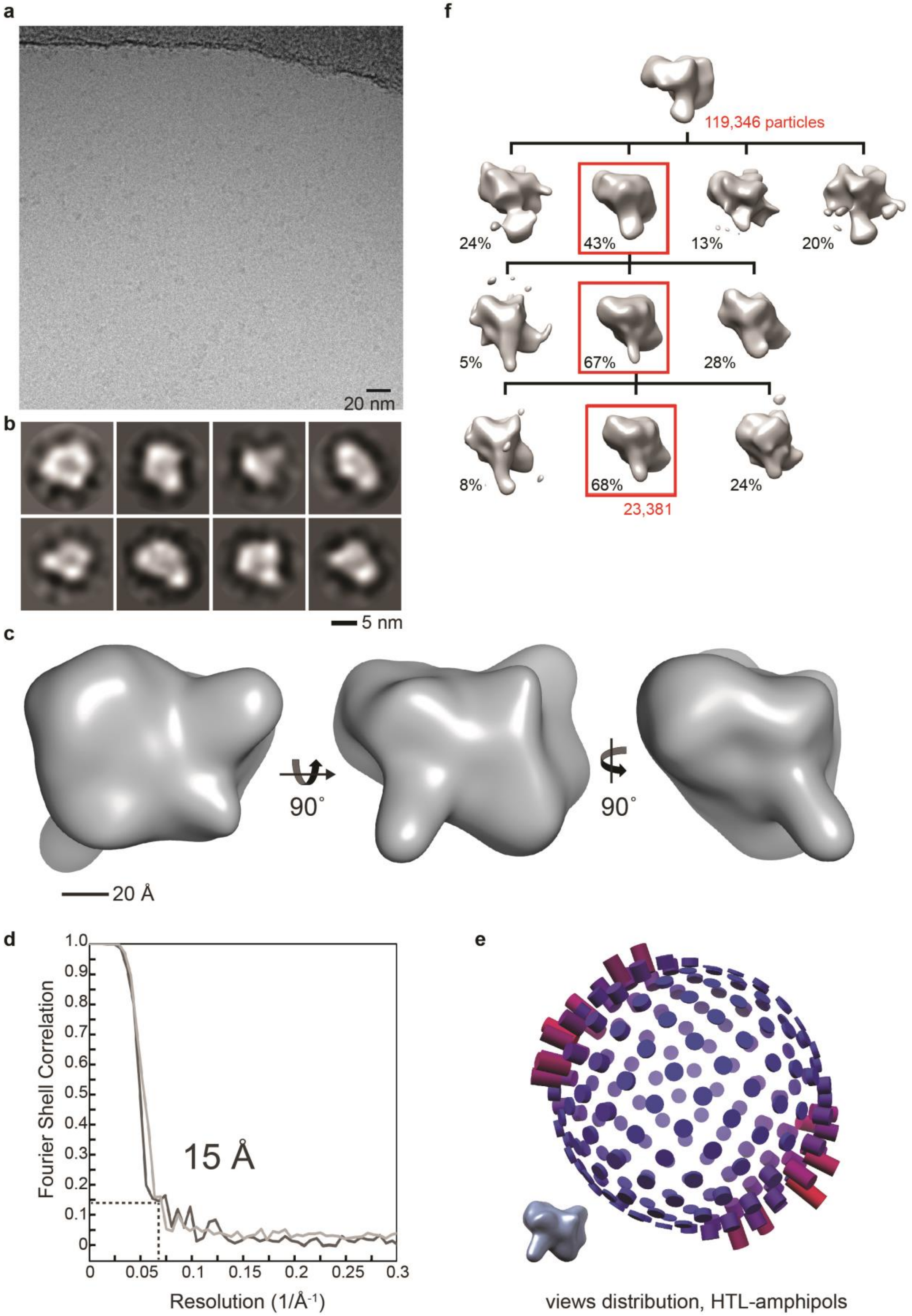
a
20 nm
b
5 nm
c
90˚
90˚
20 Å
d
Fourier Shell Correlation
15 Å
Resolution (1/Å-1)
e
views distribution, HTL-amphipols
f
119,346 particles
24%
43%
13%
20%
5%
67%
28%
8%
68%
24%
23,381

Figure S5. Cryo-EM of native HTL-amphipols. a, Original micrograph of HTL-amphipols embedded in vitreous ice. **b,** Reference-free 2D class-averages of the cryo-EM data from Relion [5]. **c,** Cryo-EM reconstruction of HTL in amphipols sample displayed in a top (left), front (middle) and side view (right). **d,** FSC curves of the cryo-EM HTL structure determined using the 'gold standard method' [2], before (light grey) and after (dark grey) high-resolution noise substitution [3]. FSC=0.143 [4] indicates a resolution of 15Å. **e,** Euler angle distribution of particles used for calculating the HTL cryo-EM structure (23,381 particles; angular step 7.5 degrees). The height of the cylinders represents the number of particles. **f,** The HTL-amphipols dataset (119,346 particles) was split randomly into four parts. The reconstruction containing most particles (43%) was further sorted computationally until a homogeneous particle pool was obtained which could not be split further. The resulting cryo-EM reconstruction was subjected to maximum likelihood 3D refinement until convergence using Relion [5].

a

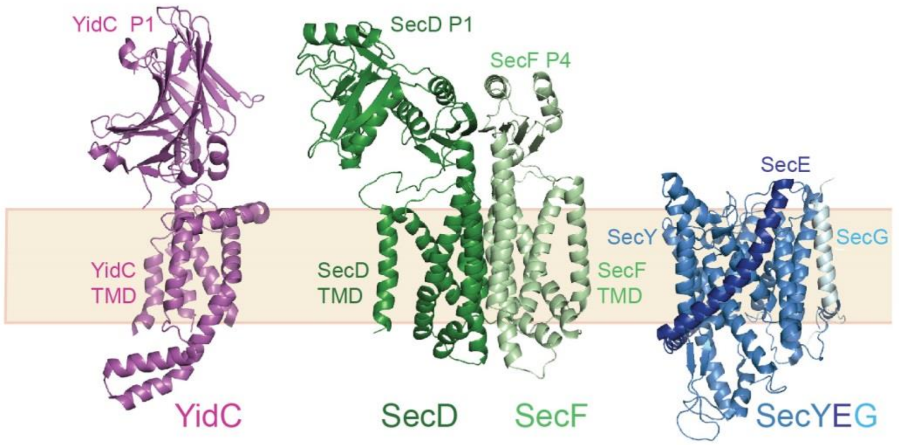

b

Protein	PDB ID	Molecular weight of the protein in the model (kDa)	Molecular weight of the *E. coli* protein / domain (kDa)	% of the quasi-atomic model
SecY	3DIN	46.3	48.5	95.5
SecE		6.1	13.6	44.7
SecG		6.9	11.4	60.7
SecD TMD	3AQP	20.3	21.3	95.3
SecD P1		25.8	45.3	57.0
SecF TMD		21.2	25.6	82.8
SecF P4		9.8	9.8	100.0
YajC	Not modeled		12.2	-
YidC	3WVF	53.2	62.2	85.5
**Total**		**189.6**	**249.9**	**75.9**

Figure S6. Summary of the structural information on HTL subunits and subassemblies used for homology modelling of the *E. coli* HTL subunits. a, Crystal structures used to generate the HTL atomic model. **b,** Table listing the available structural information for HTL subassemblies used to generate the HTL atomic model. PDB ID: Protein Data Bank accession code, TMD: transmembrane domain, P1/P4: periplasmic domains.

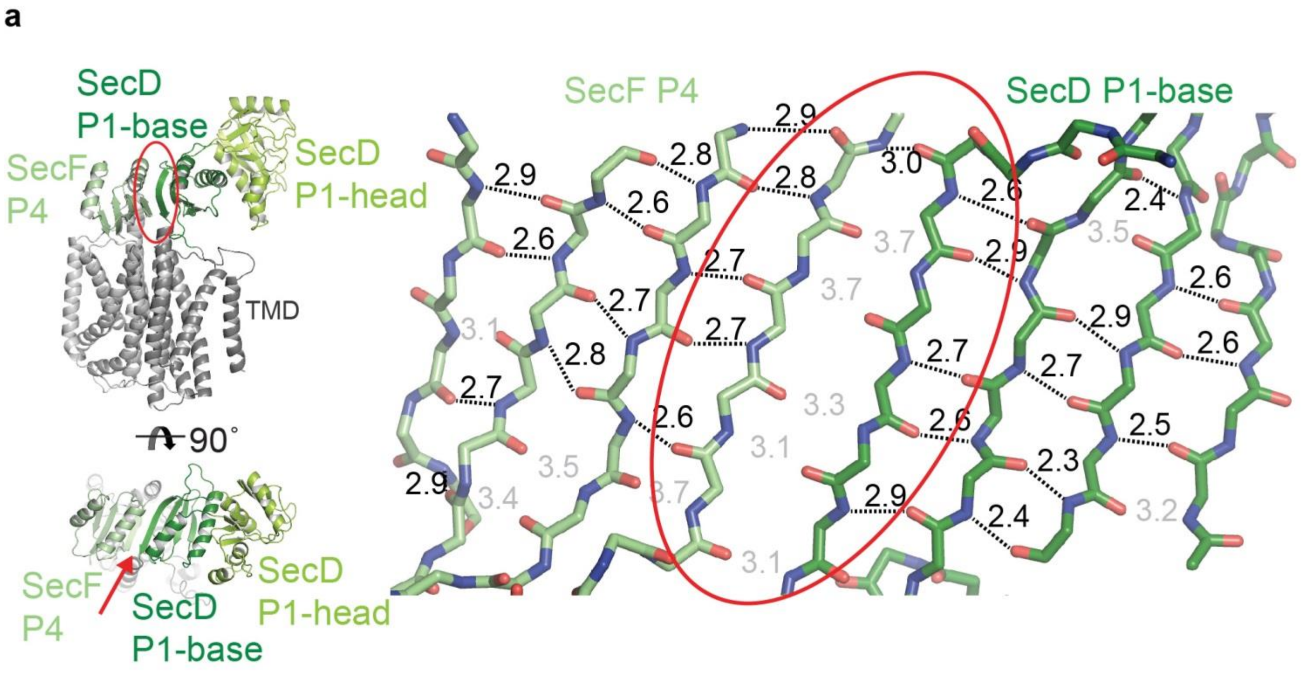
a
SecD
P1-base
SecF
P4
SecD
P1-head
TMD
90˚
SecF
P4
SecD
P1-base
SecD
P1-head
SecF P4
SecD P1-base
2.9
2.8
3.0
2.6
2.8
2.4
2.9
2.6
3.5
2.6
3.7
2.9
2.7
3.7
2.6
2.7
2.9
3.1
2.7
2.6
2.8
2.7
2.7
2.7
3.3
2.6
2.5
2.6
2.6
3.1
2.3
3.5
2.9
3.7
2.9
2.4
3.4
3.2
3.1

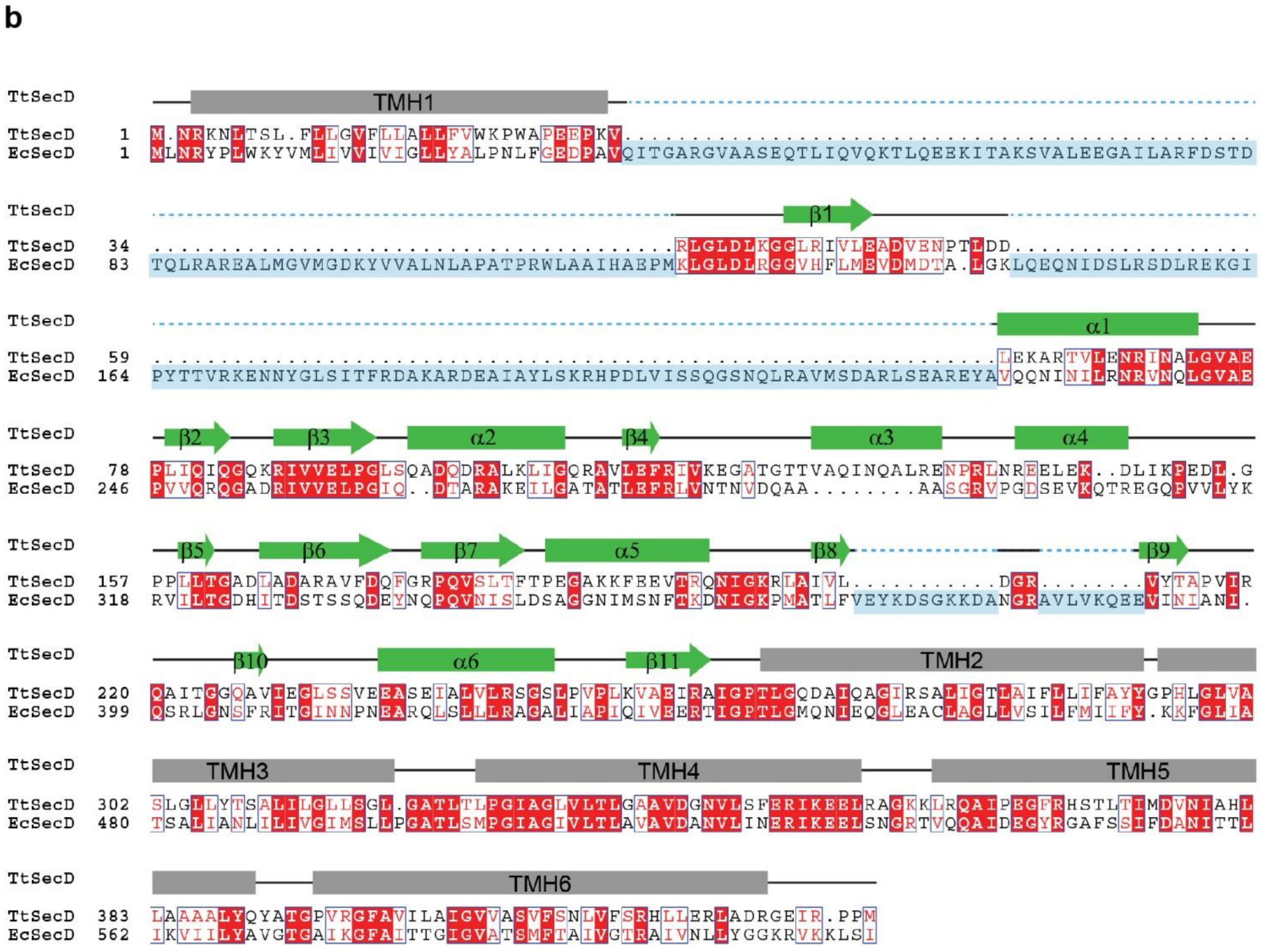
b
TtSecD TMH1
TtSecD 1 M.NRKNLTSL.FLLGVFLLALLFVWKPWAPEEPKV...............................
EcSecD 1 MLNRYPLWKYVMLIVVIVIGLLYALPNLFGEDPAVQITGARGVAASEQTLIQVQKTLQEEKITAKSVALEEGAILARFDSTD
TtSecD β1
TtSecD 34 ..................................RLGLDLKGGLRIVLEADVENPTLDD...............
EcSecD 83 TQLRAREALMGVMGDKYVVALNLAPATPRWLAAIHAEPMKLGLDLRGGVHFLMEVDMDTA.LGKLQEQNIDSLRSDLREKGI
TtSecD α1
TtSecD 59 ....LEKARTVLENRINALGVAE
EcSecD 164 PYTTVRKENNYGLSITFRDAKARDEAIAYLSKRHPDLVISSQGSNQLRAVMSDARLSEAREYAVQQNINILRNRVNQLGVAE
TtSecD β2 β3 α2 β4 α3 α4
TtSecD 78 PLIQIQGQKRIVVELPGLSQADQDRALKLIGQRAVLEFRIVKEGATGTTVAQINQALRENPRLNREELEK..DLIKPEDL.G
EcSecD 246 PVVQRQGADRIVVELPGIQ..DTARAKEILGATATLEFRLVNTNVDQAA........AASGRVPGDSEVKQTREGQPVVLYK
TtSecD β5 β6 β7 α5 β8 β9
TtSecD 157 PPLLTGADLADARAVFDQFGRPQVSLTFTPEGAKKFEEVTRQNIGKRLAIVL...........DGR........VYTAPVIR
EcSecD 318 RVILTGDHITDSTSSQDEYNQPQVNISLDSAGGNIMSNFTKDNIGKPMATLFVEYKDSGKKDANGRAVLVKQEEVINIANI.
TtSecD β10 α6 β11 TMH2
TtSecD 220 QAITGGQAVIEGLSSVEEASEIALVLRSGSLPVPLKVAEIRAIGPTLGQDAIQAGIRSALIGTLAIFLLIFAYYGPHLGLVA
EcSecD 399 QSRLGNSFRITGINNPNEARQLSLLLRAGALIAPIQIVEERTIGPTLGMQNIEQGLEACLAGLLVSILFMIIFY.KKFGLIA
TtSecD TMH3 TMH4 TMH5
TtSecD 302 SLGLLYTSALILGLLSGL.GATLTLPGIAGLVLTLGAAVDGNVLSFERIKEELRAGKKLRQAIPEGFRHSTLTIMDVNIAHL
EcSecD 480 TSALIANLILIVGIMSLLPGATLSMPGIAGIVLTLAVAVDANVLINERIKEELSNGRTVQQAIDEGYRGAFSSIFDANITTL
TtSecD TMH6
TtSecD 383 LAAAALYQYATGPVRGFAVILAIGVVASVFSNLVFSRHLLERLADRGEIR.PPM
EcSecD 562 IKVIILYAVGTGAIKGFAITTGIGVATSMFTAIVGTRAIVNLLYGGKRVKKLSI

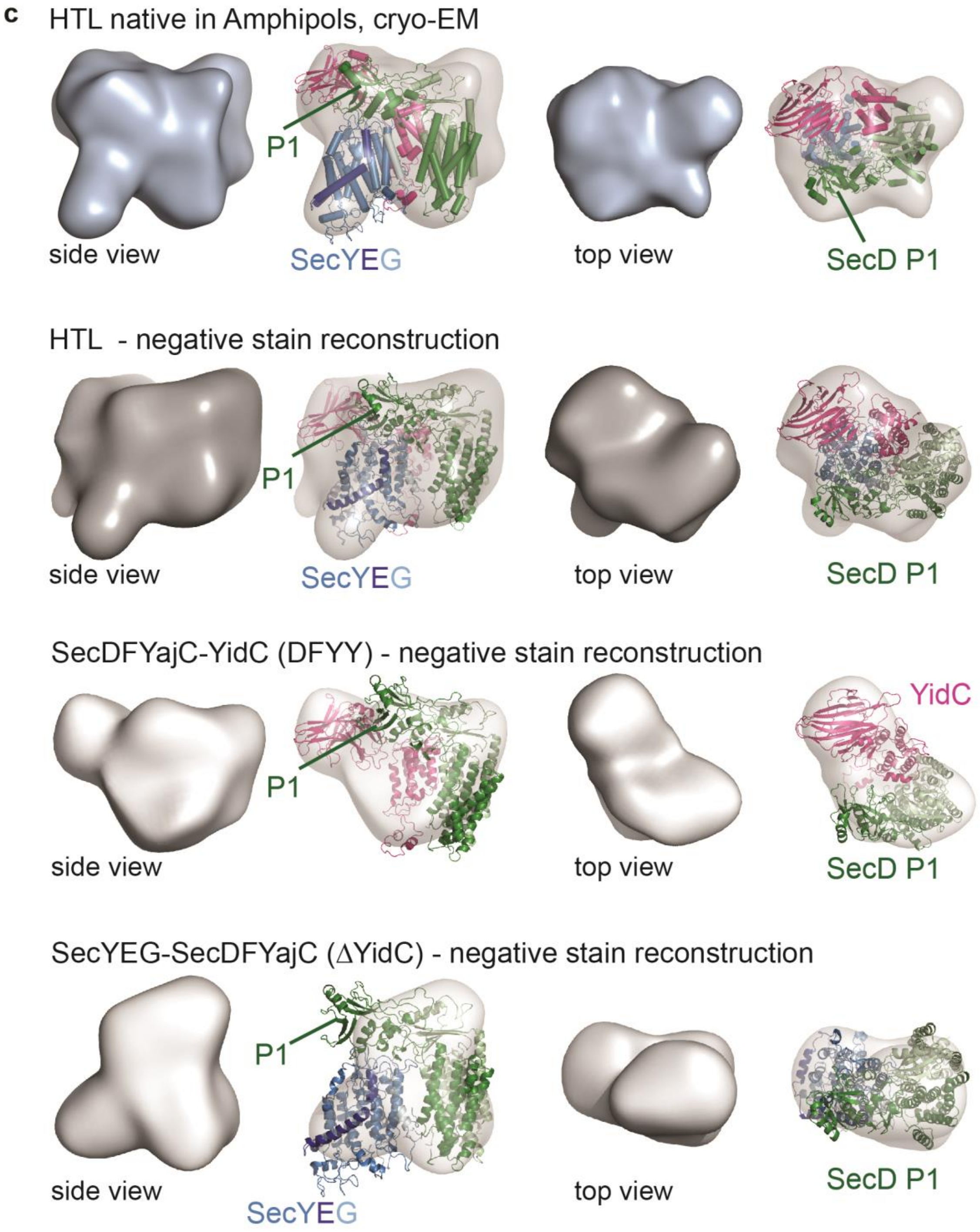

Figure S7. Conformational flexibility of the SecD P1-domain. a, The crystal structure of *Th. thermophilus* SecDF [6] indicates a continuous β-sheet between the SecD P1-base domain and SecF P4-domain (left). At the interface between SecD and SecF (encircled in red) the distances between the C=O groups and N-H groups are longer compared to intra-domain distances (right). **b,** Sequence alignment of SecD from *Th. thermophilus* and *E. coli*. The global alignment of the different SecD protein sequences was performed using the T-coffee package

(http://tcoffee.crg.cat/apps/tcoffee/index.html). The alignment of the sequences with the secondary structure was performed using the ESPript program (http://espript.ibcp.fr/ESPript/ESPript/). Identical residues are shown as white letters in a red box, similar residues as red letters in a white box, variable residues as black letters, and dots represent gaps in the alignment. The parts of the *E. coli* sequence highlighted in light blue represent the additional residues present in the P1-domain compared to the *Th. thermophilus* sequence. The secondary structure based on the available crystal structure (PDB code: 3AQP [6]) is shown on top of the alignment: α : α -helix (grey); β : β -sheet (green). **c,** Placement of HTL model subunits into the cryo-EM reconstruction of HTL-amphipols and into the negative stain reconstructions of HTL, DFYY and ΔYidC. The fitting clearly shows that our HTL model with the rotated SecD periplasmic domain is compatible with all EM reconstructions of SecDF-containing complexes.

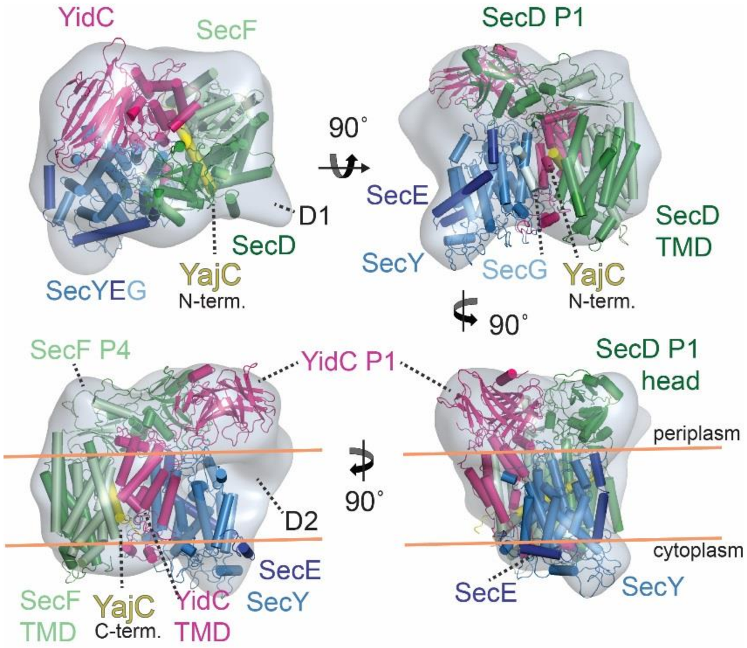

Figure S8: Fitting of crystal structures (YidC) and homology models of SecYEG (blue), SecDF (green) and YajC (yellow) into HTL density (transparent grey), shown in a top (top left), front (top right) and two side views (below). Periplasmic domains of YidC, SecD and SecF are labelled P1 and P4 respectively. Unaccounted density is labelled D1 and D2.

The position of the transmembrane helix of YajC has been modelled based on the homology between AcrB and SecDF which both belong to the resistance-nodulation-cell division superfamily. The contact surface between AcrB and AcrZ which modulates AcrB's function is conserved in SecDF. Du *et al.* suggested that SecDF binds YajC at the same position as in the AcrB-AcrZ crystal structure [7]. Like AcrZ, YajC may induce a rotation/twist in SecDF and play an allosteric role in modulation of SecDF's activity [7].

Supplementary Tables

Table S1: Summary of SANS data collected for the native and cross-linked HTL complexes. I(0) and R_g were determined from Guinier analysis of the data (Fig. S2). The linear fit of the data was valid in the q-range of q smaller than q_{max}. The units of I(0) were calibrated with respect to 0.100 cm of water measured under identical conditions. The R_g for the cross-linked HTL in 20% D_2O for was omitted from analysis, as the contrast was too low for a meaningful measurement.

D_2O	Protein concentration (mg/ml)	I(0)	Error in I(0)	R_g (Å)	$q_{max}.R_g$
**native HTL**					
H_2O	3.80	1.10	0.03	68.9 ± 1.9	1.54
16%D_2O	2.52	0.130	0.006	69.0 ± 1.7	2.53
36%D_2O	2.80	0.342	0.006	44.4 ± 1.2	1.22
70%D_2O	3.12	4.41	0.06	54.4 ± 1.1	1.21
**cross-linked HTL**					
H_2O	0.90	0.433	0.007	42.5 ± 1.2	1.14
10%D_2O	0.70	0.092	0.006	46.8 ± 4.0	1.26
20%D_2O	0.72	0.015	0.010	54.1 ± 33.9	1.45
60%D_2O	0.87	0.843	0.006	31.3 ± 0.7	0.84
90%D_2O	1.44	4.64	0.02	35.0 ± 0.4	0.94

Table S2: List of interactions of SecYEG, SecDF, YajC and YidC.

Protein 1	Protein 2	Involved Residues	Experimental Approach	Supplementary References
YidC	SecYEG	n/a	Co-immunoprecipitation	(8)
YidC	SecYEG	TM domain	Synthetic lethal screen & complementation assays	(9)
YidC	SecY	Residues in the SecY lateral gate (TM2b, TM3, TM7 & TM8)	Cross-linking studies	(10)
YidC	SecD YajC SecG	YidC 249 [located in the P1-domain]	Cross-linking studies	(10)
YidC	SecF YajC SecY	YidC 540 [cytoplasmic C-terminus]	Cross-linking studies	(10)
YidC	SecF	YidC P1 [residues 215-265]	Co-immunoprecipitation	(11)
YajC	SecDF	n/a	Co-immunoprecipitation	(12)
YajC	SecYEG	n/a	Co-immunoprecipitation	(12)
SecY	SecD and YidC	n/a	Cross-linking studies	(13)
SecE	SecD YidC	n/a	Cross-linking studies	(13)
SecG	SecD YidC	n/a	Cross-linking studies	(13)

n/a: information not available.

Supplementary References

1. Li, G.W., Burkhardt, D., Gross, C. & Weissman, J.S. Quantifying absolute protein synthesis rates reveals principles underlying allocation of cellular resources. *Cell* **157**, 624-635 (2014).
2. Scheres, S.H. & Chen, S. Prevention of overfitting in cryo-EM structure determination. *Nat Methods* **9**, 853-854 (2012).
3. Chen, S. *et al.* High-resolution noise substitution to measure overfitting and validate resolution in 3D structure determination by single particle electron cryomicroscopy. *Ultramicroscopy* **135**, 24-35 (2013).
4. Rosenthal, P.B. & Henderson, R. Optimal determination of particle orientation, absolute hand, and contrast loss in single-particle electron cryomicroscopy. *J Mol Biol* **333**, 721-745 (2003).
5. Scheres, S.H. RELION: implementation of a Bayesian approach to cryo-EM structure determination. *J Struct Biol* **180**, 519-530 (2012).
6. Tsukazaki, T. *et al.* Structure and function of a membrane component SecDF that enhances protein export. *Nature* **474**, 235-238 (2011).
7. Du, D. *et al.* Structure of the AcrAB-TolC multidrug efflux pump. *Nature* **509**, 512-515 (2014).
8. Scotti, P.A. *et al.* YidC, the *Escherichia coli* homologue of mitochondrial Oxa1p, is a component of the Sec translocase. *EMBO J* **19**, 542-549 (2000).
9. Li, Z., Boyd, D., Reindl, M. & Goldberg, M.B. Identification of YidC residues that define interactions with the Sec Apparatus. *J Bacteriol* **196**, 367-377 (2014).
10. Sachelaru, I. *et al.* YidC occupies the lateral gate of the SecYEG translocon and is sequentially displaced by a nascent membrane protein. *J Biol Chem* **288**, 16295-16307 (2013).
11. Xie, K., Kiefer, D., Nagler, G., Dalbey, R.E. & Kuhn, A. Different regions of the nonconserved largeperiplasmic domain of *Escherichia coli* YidC are involved in the SecF interaction and membrane insertase activity. *Biochemistry* **45**, 13401-13408 (2006).
11. Xie, K., Kiefer, D., Nagler, G., Dalbey, R.E. & Kuhn, A. Different regions of the nonconserved large periplasmic domain of *Escherichia coli* YidC are involved in the SecF interaction and membrane insertase activity. *Biochemistry* **45**, 13401-13408 (2006).
12. Duong, F. & Wickner, W. Distinct catalytic roles of the SecYE, SecG and SecDFyajC subunits of preprotein translocase holoenzyme. *EMBO J* **16**, 2756-2768 (1997).
13. Schulze, R.J. *et al.* Membrane protein insertion and proton-motive-force-dependent secretion through the bacterial holo-translocon SecYEG-SecDF-YajC-YidC. *Proc Natl Acad Sci USA* **111**, 48844-48849 (2014).
